# The Interleukin 3 Gene (IL3) Contributes to Human Brain Volume Variation by Regulating Proliferation and Survival of Neural Progenitors

**DOI:** 10.1371/journal.pone.0050375

**Published:** 2012-11-30

**Authors:** Xiong-jian Luo, Ming Li, Liang Huang, Kwangsik Nho, Min Deng, Qiang Chen, Daniel R. Weinberger, Alejandro Arias Vasquez, Mark Rijpkema, Venkata S. Mattay, Andrew J. Saykin, Li Shen, Guillén Fernández, Barbara Franke, Jing-chun Chen, Xiang-ning Chen, Jin-kai Wang, Xiao Xiao, Xue-bin Qi, Kun Xiang, Ying-Mei Peng, Xiang-yu Cao, Yi Li, Xiao-dong Shi, for the Alzheimer’s Disease Neuroimaging Initiative, Lin Gan, Bing Su

**Affiliations:** 1 State Key Laboratory of Genetic Resources and Evolution, Kunming Institute of Zoology, Chinese Academy of Sciences, Kunming, Yunnan, China; 2 University of Rochester Flaum Eye Institute, University of Rochester, Rochester, New York, United States of America; 3 Gannan Medical University, Ganzhou, Jiangxi, China; 4 Center for Neuroimaging, Department of Radiology and Imaging Sciences, Indiana University School of Medicine, Indianapolis, Indiana, United States of America; 5 Center for Computational Biology and Bioinformatics, Indiana University School of Medicine, Indianapolis, Indiana, United States of America; 6 Regenstrief Institute, Indianapolis, Indiana, United States of America; 7 Department of Medical and Molecular Genetics, Indiana University School of Medicine, Indianapolis, Indiana, United States of America; 8 Genes, Cognition and Psychosis Program, National Institute of Mental Health, the National Institutes of Health, Bethesda, Maryland, United States of America; 9 Department of Genetics, Radboud University Nijmegen Medical Centre, Nijmegen, The Netherlands; 10 Department of Psychiatry, Donders Institute for Brain, Cognition and Behaviour, Radboud University Nijmegen Medical Centre, Nijmegen, The Netherlands; 11 Donders Institute for Brain, Cognition and Behaviour, Donders Centre for Cognitive Neuroimaging, Radboud University Nijmegen, Nijmegen, The Netherlands; 12 Department of Cognitive Neuroscience, Donders Institute for Brain, Cognition and Behaviour, Radboud University Nijmegen Medical Centre, Nijmegen, The Netherlands; 13 Virginia Institute for Psychiatric and Behavioral Genetics, and Department of Psychiatry, Virginia Commonwealth University, Richmond, Virginia, United States of America; 14 Biological Resources and Environmental Science College, Qujing Normal University, Qujing, Yunnan, China; 15 College of Life and Environmental Sciences, Hangzhou Normal University, Hangzhou, Zhejiang, China; Emory University, United States of America

## Abstract

One of the most significant evolutionary changes underlying the highly developed cognitive abilities of humans is the greatly enlarged brain volume. In addition to being far greater than in most other species, the volume of the human brain exhibits extensive variation and distinct sexual dimorphism in the general population. However, little is known about the genetic mechanisms underlying normal variation as well as the observed sex difference in human brain volume. Here we show that interleukin-3 (IL3) is strongly associated with brain volume variation in four genetically divergent populations. We identified a sequence polymorphism (rs31480) in the IL3 promoter which alters the expression of IL3 by affecting the binding affinity of transcription factor SP1. Further analysis indicated that IL3 and its receptors are continuously expressed in the developing mouse brain, reaching highest levels at postnatal day 1–4. Furthermore, we found IL3 receptor alpha (IL3RA) was mainly expressed in neural progenitors and neurons, and IL3 could promote proliferation and survival of the neural progenitors. The expression level of IL3 thus played pivotal roles in the expansion and maintenance of the neural progenitor pool and the number of surviving neurons. Moreover, we found that IL3 activated both estrogen receptors, but estrogen didn’t directly regulate the expression of IL3. Our results demonstrate that genetic variation in the IL3 promoter regulates human brain volume and reveals novel roles of IL3 in regulating brain development.

## Introduction

The greatly expanded brain size and highly developed cognitive abilities are the most significant features that set humans apart from other species. In addition, human brain size is also highly variable in the general population, ranging from 981 ml to 1,795 ml (1,462 ml in males and 1,266 ml in females, on average) [1]. Recent imaging studies using MRI techniques have revealed a high heritability (0.82–0.87) of brain volume and its correlation with general intelligence [2], [3], working memory, perceptual organization and processing speed [4]. It has been well established that the enlarged brain volume is the basis of our unique cognitive capacity, and a reduction of brain volume has been reported in several brain diseases such as schizophrenia [5] and Attention-Deficit/Hyperactivity Disorder (ADHD) [6]. As a complex quantitative trait with high heritability, brain volume is likely regulated by many genes. But so far only a handful of such genes have been reported by studying patients with rare brain developmental defects, *e.g.* microcephaly [7]. Though the reported microcephalin genes are important in explaining the enlarged human brain during evolution [8], recent studies have indicated that they only account for a small part of brain volume variation in the general population [9], [10]. Fortunately, recent genome wide association studies have identified several promising loci significantly associated with intracranial volume and head circumference [11]–[13]. Nevertheless, all of these studies were performed only in populations of European ancestry and some of the variants (e.g., rs7890687 and rs9915547) identified in these GWAS were fixed (monomorphic) in Chinese population, suggesting that additional genes/variants may modulate brain volume variation in Chinese population.

Additionally, the genetic dissection of schizophrenia (SCZ), a common mental disorder with high heritability provides opportunities to identify genes associated with brain volume variation since SCZ patients have decreased total brain volume compared to normal controls [5], [14], [15]. This is consistent with the hypothesis that the pathogenesis of SCZ is related to abnormal brain development [16]. Though numerous linkage and association studies, especially recent genome wide association studies have identified many loci significantly associated with schizophrenia [17]–[22], the etiology of schizophrenia remains poorly understood. Among the hypotheses that explain the etiology of schizophrenia, the neurodevelopmental hypothesis [23] has been supported by the majority of the published data. This hypothesis predicts that a disruption of brain development during early life underlies the later emergence of psychosis during adolescence or early adulthood. These evidences indicate that schizophrenia susceptibility genes may regulate the unique features of human brain development and dysfunction of these genes likely disrupted the normal development of brain, which eventually lead to schizophrenia susceptibility. In fact, recent studies demonstrated that some schizophrenia susceptibility genes do regulate brain development [24], [25]. In light of these findings, we hypothesize that schizophrenia susceptibility genes may regulate brain development and affect the total brain volume.

To detect the relationship between schizophrenia susceptibility genes and brain volume, we earlier systematically studied the genetic association between schizophrenia susceptibility genes and brain volume variation in a large cohort of healthy subjects. This led to identification of a highly significant chromosomal region, 5q23.2–33.1, a region that has been well studied and shown strong association with SCZ in multiple world populations [26]–[32]. Recently, Chen *et al*. systematically studied this region by using a large sample (N = 3,422, including case-control and family-based samples) and dense SNP markers. They found haplotypes spanning SPEC2, PDZ-GEF2, LOC728637, and ACSL6 were significantly associated with schizophrenia in five independent samples [33], [34]. We further replicated the associations in a Chinese sample [35]. Collectively, these consistent results strongly suggested genetic variants near these four genes (SPEC2, PDZ-GEF2, LOC728637, and ACSL6) may contribute to schizophrenia susceptibility and brain development.

## Results

### Interleukin-3 is Strongly Associated with Brain Volume Variation in Chinese

**Figure 1:**
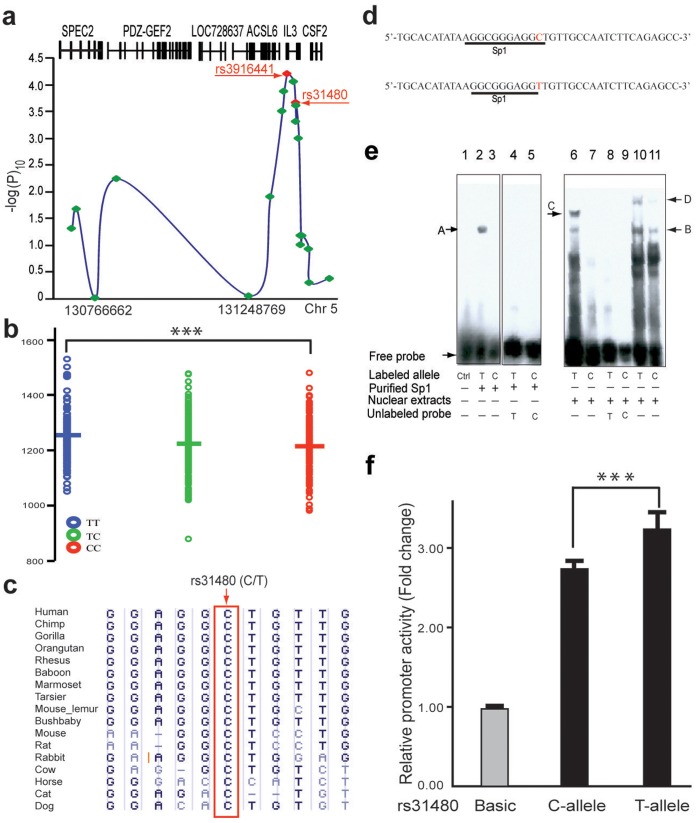
Genetic association of the 5q23.2–33 SNPs with brain volume and impacts of promoter SNP (rs31480) on the expression of IL3. **(a)** The distribution of the –logP of the 20 SNPs tested across the 5q23.3–33.1 region (middle panel). The locations of the six known coding genes are displayed. **(b)** The brain volume distributions of the three genotypes at rs31480, on average, TT genotype carriers have a brain volume of 1257 ml and CC genotypes have 1216 ml (***P<0.001, two tailed Student’s *t-test*). **(c)** C allele of rs31480 is completely conserved across a variety of species. **(d)** The oligonucleotides for testing the binding activity of SP1. The predicted binding sequence is underlined containing the rs31480 variation site (red). **(e)** The result of electrophoretic mobility shift assay, showing that the probe containing T allele can bind SP1 (Lane 2) but the C allele cannot (Lane 3). Similar results were obtained using HeLa or MCF7 nuclear extracts (Lane 6 and 7, 10 and 11). Competition experiments using a 100-fold excess of unlabeled probe (Lane 4 and 5, 8 and 9) confirm the specificity of the probe. Binding to the unknown protein/complex was also observed (Figure 1e, arrow C and D), again, the probe containing T allele showed stronger binding than C allele. **(f)** Assays of promoter activities by relative luciferase expression in HeLa, construct with T allele has significant higher expression activity than C allele. Values of relative luciferase activity are expressed as mean ± s.d. (results of three independent experiments, each containing three replicates). ***P<0.001 (one tailed Student’s *t-test*).

For the initial analyses in Chinese population, we performed a genetic screening to detect the association of cranial volume (the approximate of brain volume, which is highly correlated with brain volume [36], [37]) with sequence variations located in the 5q23.2–33.1 region. The cranial volumes of 1,013 healthy individuals (460 males and 553 females) were measured (see methods), followed by genotyping of 20 single nucleotide polymorphisms (SNPs) in the 5q23.2–33.1 region spanning about 809 kb. To test whether schizophrenia susceptibility variants in 5q23.2–33.1 are associated with brain volume, we initially genotyped 8 tagging SNPs covering the four genes (SPEC2, PDZ-GEF2, LOC728637, and ACSL6). The single SNP association was conducted using linear regression under an additive model and the p-values were obtained by the Wald test as implemented in PLINK [38]. The results showed that six of these 8 SNPs were significantly associated with cranial volume in females, but not in males (**Table S1**). For fine-scale mapping, we genotyped another 12 SNPs and identified a sharp signal in the region containing IL3, showing a strong female-specific association with cranial volume (**Fig. 1a** **and Table S1**). Among the 7 highly significant SNPs (-logP>3.3) covering IL3, one was located in exon 1 (rs40401, Ser to Pro), one in intron 2 (rs31481), one in the promoter (rs31480), and four in the upstream region (rs3914025, rs3916441, rs31400 and rs3846726) (**Fig. 1a**), clearly implicating IL3 as the responsible gene. The associations between these 7 SNPs and brain volume were still highly significant (corrected p<0.01) even using the most stringent Bonferroni correction for multiple testing (**Table S1**). Further haplotype analysis combining the 7 SNPs indicated strong linkage disequilibrium (LD) among the SNPs (**Fig. S1a**) with only two major haplotypes, one showing positive association (P = 4×10^−5^), the other showing negative association (P = 8×10^−4^) with cranial volume in females (**Table S2**). None of the described associations in females were observed in males (**Table S3**), implying that the association of IL3 with brain volume is sex-specific. To capture missing common SNPs, we re-sequenced the IL3 gene region (4 kb) in 150 randomly selected Chinese individuals and found no additional SNPs.

### Replication of the Association between IL3 and Brain Volume Variation in Europeans

To confirm our initial findings from the Chinese population, we conducted a replication analysis in three independent samples of European ancestry, for which total brain volume had been determined based on magnetic resonance imaging (MRI). For the seven SNPs showing strong association in Chinese sample, two SNPs in different LD regions in Europeans (rs3916441 and rs40401, **Fig. S1**) were included in the replication analysis. Only the healthy controls of these samples were used. We found that the most significant SNP in Chinese, rs3916441, was also significantly associated with total brain volume in the BIG (Dutch Brain Imaging Genetics study) sample (p = 3.5×10^−4^; n = 486) (**Table 1** **)**. In CBDB/NIMH (Clinical Brain Disorders Branch/National Institute of Mental Health Sibling Study) sample (n = 188), rs3916441 also showed a trend of association (p = 0.0516) (**Table 1**). We noticed the female specific association of rs3916441 with brain volume in Chinese was not the situation in European samples. Interestingly, rs3916441 was also significantly associated with total gray matter volume in the CBDB/NIMH and BIG samples (**Table 2**), which may point towards a mechanism explaining the effects of IL3 on brain structure.

**Table 1:** Replication of the most significantly associated SNPs in genetically divergent populations.

SNP	Polymorphism	Replication samples								
		ADNI sample(Healthy controls)			CBDB/NIMH sample(Healthy controls)			BIG sample (3.0 T)(Healthy controls)		
		P value (Total brain volume)			P value (Total brain volume)			P value (Total brain volume)		
		All(n = 204)	Male(n = 110)	Female(n = 94)	All(n = 188)	Male(n = 89)	Female(n = 99)	All(n = 486)	Male(n = 194)	Female(n = 292)
rs3916441	C/T	0.697	**0.0467**	0.164	**0.0516**	**0.0416**	0.462	**3.5×10** ^−**4**^	**3.08×10** ^−**4**^	0.130
rs40401	G/A	0.147	0.261	0.467	0.703	0.580	NA	NA	NA	NA

NA: Not available.

**Table 2:** Association of rs3916441 with gray matter and white matter in genetically divergent populations.

SNP	Polymorphism	Replication samples								
		CBDB/NIMH sample(Healthy controls)			BIG sample (3.0 T) (Healthy controls)					
		P value (Total gray matter volume)			P value (Total gray matter volume)			P value (Total white matter volume)		
		All(n = 89)	Male(n = 99)	Female(n = 486)	All(n = 486)	Male(n = 194)	Female(n = 292)	All(n = 486)	Male(n = 194)	Female(n = 292)
rs3916441	C/T	**0.0228**	0.128	0.110	**0.001**	**0.001**	0.178	**0.002**	**0.004**	0.138
rs40401	G/A	0.171	0.128	NA	NA	NA	NA	NA	NA	NA

NA: Not available.

### Impacts of rs31480 on Transcription Factor Binding and IL3 Expression

To capture the causal variants of IL3 in Chinese population, we performed bioinformatics analysis for the 7 highly significant SNPs according to their genomic locations and allelic differences in transcription factor binding affinities, and we found SNP rs31480 showing potential functional effects. rs31480 is located in the IL3 promoter (−16 bp upstream of the transcription start site (TSS), **Fig. S2**), within a putative binding site of the transcription factor SP1. Interestingly, there is a significant difference of 41 ml in the average cranial volume between individuals carrying the two homozygotes at rs31480 (1,257 ml for TT carriers and 1,216 ml for CC carriers, p = 4.7 ×10^−4^, two tailed student t-test) (**Fig. 1b** **and Table S4**), suggesting that rs31480 could regulate brain volume variation. In addition, we noticed the C allele (ancestral allele, determined by comparison with the chimpanzee homologous sequence) of rs31480 is completely conserved across a wide variety of species (**Fig. 1c** **and Fig. S2**), also suggesting a functional conservation of rs31480. The T allele (derived allele) is prevalent in East Asian populations (0.556 in Chinese and 0.568 in Japanese), but relatively rare in Europeans (0.198) and Africans (0.136) (http://www.hapmap.org).

The C to T change at rs31480 could change the binding affinity of SP1 and influence the expression of IL3. Electrophoretic mobility shift assay (EMSA) with purified recombinant human SP1 protein (**Fig. 1d**) showed that SP1 binds to the sequence containing the T allele (**Fig. 1e**, lane 2) but not the C allele (**Fig. 1e**, lane 3). Similar results were observed when using HeLa (**Fig. 1e**, lane 6 and 7) and MCF-7 (**Fig. 1e**, lane 10 and 11) cell nuclear extracts as the source of SP1 protein (**Fig. 1e**). Finally, competition experiments using unlabeled oligonucleotides corroborated the SP1 binding specificity to the T allele (**Fig. 1e**, lane 4 and 5, lane 8 and 9). These data suggest that rs31480 has an “on” or “off” effect on SP1 binding to the IL3 promoter.

To test whether rs31480 also influences IL3 promoter activity, we performed transactivation assays using the luciferase reporter gene. The promoter region encompassing nucleotides −436 to +164 (relative to the ATG start codon at +1) of IL3 was amplified by PCR from genomic DNA of two individuals homozygous with respect to the corresponding genotypes (TT and CC) for rs31480. Sequencing analysis of the amplified promoter fragments did not detect other sequence differences except for rs31480. As shown in **Fig. 1f** and **Fig. S3a–c**, the transcriptional activity of the IL3 promoter containing the T allele was indeed significantly higher than that of the C allele in all cell lines tested (Hela, CHO, SK-N-SH, and COS-7). The T allele of rs31480 thus enhances the IL3 promoter activity through the binding of transcription factor SP1. For the most significant SNP rs3916441, since our functional prediction analysis did not give any hint for the functional role of this SNP, whether it plays any functional role for IL3 is yet to be determined.

It should be noted that, rs31480 was not significantly associated with brain volume in the BIG sample of Europeans, and it was not available in the ANDI and CBDB/NIMH samples. Another SNP rs40401 in high linkage with rs31480 was also not significant in these samples. The differences in association for this SNP (rs31480) are likely due to the genetic heterogeneity between Chinese and Europeans as shown in **Fig. S1**.

### IL3 and its Receptors are Mainly Expressed in Neural Progenitors and Mature Neurons

**Figure 2:**
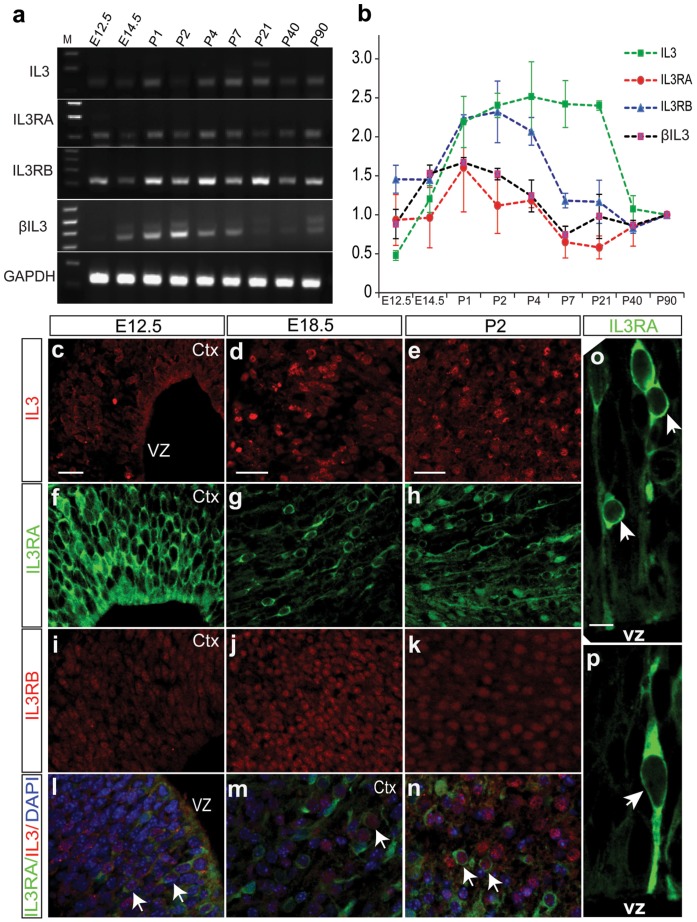
Spatiotemporal expression profiling of IL3 and its receptor in the developing mouse brain. (**a**) RT-PCR revealed the expression of IL3 and its receptor in developing mouse brain from E12.5 to adult. (**b**) Quantitative PCR showed that the expression of IL3 and its receptors peaks at P1–P4, a period with active neural proliferation and neurogenesis. Data are expressed as mean ± s.e.m. (n = 3). (**c–n**) Immunohistochemistry analysis indicated that IL3 and its receptor were expressed in the mouse brain. Co-expression of IL3 and IL3RA were detected (arrows in **l–n**), indicating the activation of IL3-mediated signaling pathways in the developing mouse brain. (**o–p**) IL3RA is expressed in radial glia (resides in ventricular zone and characterized by long radial processes, arrowhead in **o**) and migratory neurons (arrowheads in **p**). Ctx, cortex; VZ, ventricular zone. Scale bars, (c, d, e) 25 µm; O, 10 µm.

**Figure 3:**
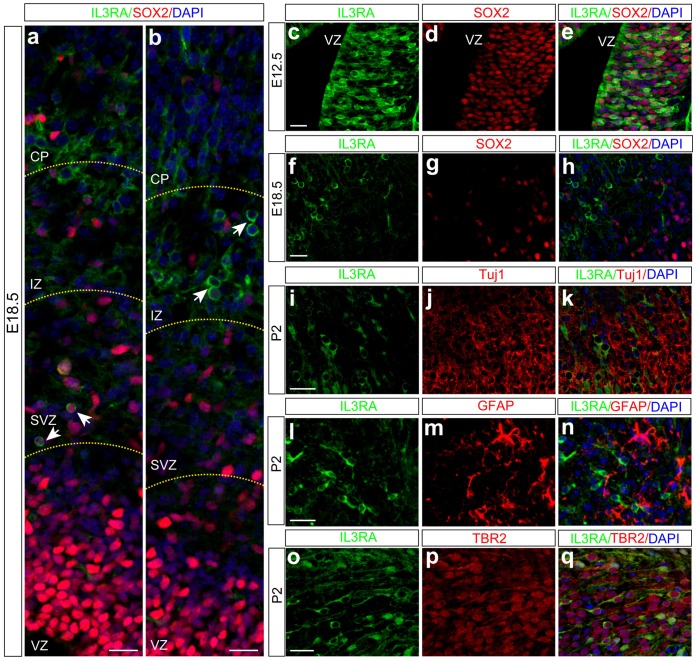
IL3RA is mainly expressed in neural progenitors and neurons. (**a, b**) Expression of IL3RA was detected in SVZ and IZ regions. Co-labeling with SOX2 showed IL3RA expression cells in SVZ are SOX2 positive, indicating these cells are neural progenitors. However, IL3RA positive cells in IZ are SOX2 negative, indicating these cells are not neural progenitors. (**c–e**) In the early stages of brain development, co-expression of IL3RA and SOX2 was found in neocortex region. With the development of the central nervous system, expression of SOX2 was down-regulated or disappeared in IL3RA positive cells (**f–h**). We also found many IL3RA positive cells were not mature neurons (**i–k**) or glial cells (**l–n**). Co-labeling with TBR2 demonstrated IL3RA positive cells are intermediate progenitor cells (IPCs) (**o–q**). VZ, ventricular zone; SVZ, subventricular zone; IZ, intermediate zone; CP, cortex plate. Scale bars, 25 µm.

IL3 exerts its biological effects through a receptor that is composed of a ligand-specific α (IL3RA) subunit and a signal transducing β subunit (IL3RB) common to IL3/IL5/GM-CSF. The mouse IL3 receptor has two distinct β subunits, one that functions only in IL3-mediated cell signaling (βIL3) and a second that is shared with IL5 and GM-CSF (IL3RB or CSF2RB). We studied the expression of IL3 and its receptors in the developing mouse brain and found that IL3 and its receptors were continuously expressed in mouse brain from embryonic day (E) 12.5 to adult life as revealed by RT-PCR (**Fig. 2a**), with a peak expression level at postnatal day (P) 1 to 4 (**Fig. 2b**), a stage with active neural proliferation and neurogenesis. We also noticed that βIL3 is only expressed from E14.5 to P7 (**Fig. 2a**), a stage accompanied by the dramatic increase of the neocortex volume [39]. Immunostaining revealed that IL3 and its receptors were mainly expressed in the neocortex region of the mouse brain (**Fig. 2c–n** and **Fig. S4** and **S5**). We also detected weak expression of IL3RA in the CA1 and CA3 regions of hippocampus (**Fig. S6**), hilus of the dentate gyrus (**Fig. S7**), and lateral septal nucleus, dorsal part (LSD) (**Fig. S8**). Compared to IL3RA, IL3RB showed higher expression in the mouse brain (**Fig. 2a, b**). It was extensively expressed in mouse brain including neocortex (**Fig. 2i–k**) and hippocampus (**Fig. S6**). Since all IL3RA positive cells also expressed IL3RB (**Fig. S6** and **S9**), we focused on IL3RA hereinafter. To characterize IL3RA-positive cells, we performed co-immunostaining and found IL3RA was expressed in SOX2- and nestin-positive neural progenitors at early developmental stage (**Fig. 3a–e**, **Fig. S4** and **S10**). As development continues, the expression of IL3RA was down-regulated in neural progenitors (**Fig. 3f–h**). After birth, expression of IL3RA was found in some Tuj1-positive neurons (**Fig. S11**). However, we noticed that many IL3RA positive cells were Tuj1-negative (**Fig. 3i–k** **, Fig. S11a–i**). Co-labeling with GFAP excluded their identity as glial cells (**Fig. 3l–n**). Further double immunostaining showed many IL3RA positive cells also weakly expressed Tbr2, a marker for intermediate progenitor cells (IPCs) (**Fig. 3o–q** and **Fig. S12**). In contrast, in addition to co-expression with IL3RA, IL3RB was expressed in neurons and glial cells (**Fig. S13**). Taken together, these results demonstrate that IL3 and its receptors are mainly expressed in neural progenitors and neurons in the developing neocortex.

### IL3 Promotes the Proliferation of Neural Progenitors

**Figure 4:**
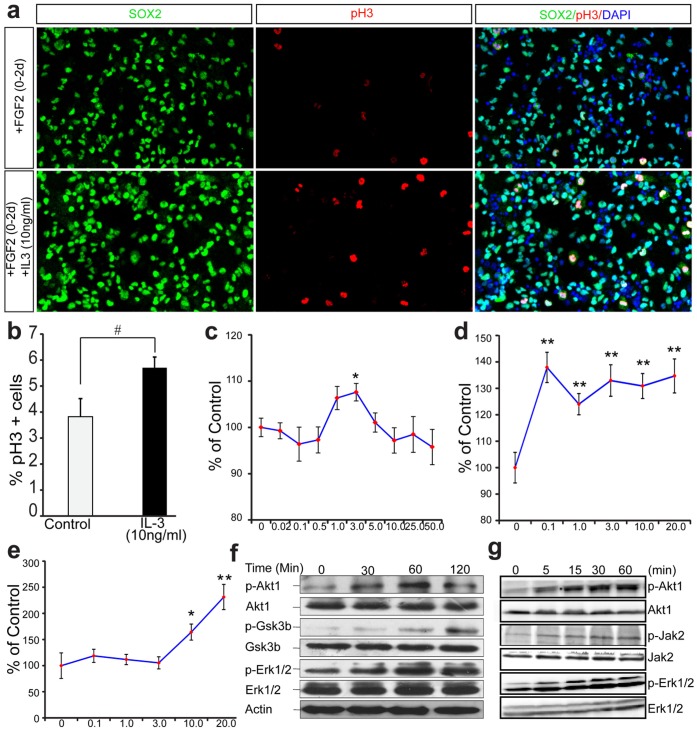
IL3 promotes proliferation and survival of neural progenitors. (**a**) IL3 promotes neural progenitor’s proliferation. (**b**) Quantification of proliferating cells (pH3+) after IL3 treatment. ^#^ *P*<0.05 (n = 4, one-tailed Student’s *t*-test). (**c**) Trophic effect of IL3 on neural progenitors. Neural progenitors from E12.5 mice were grown in the absence of any factor and in the presence of different concentrations of IL3 for 36 hours, then cell viability were determined. Note that 3.0 ng/ml IL3 could promote survival of neural progenitors significantly (n = 8 for each condition). (**d–e**) Neurotrophic effects of IL3 on neural progenitors and neurons. Neural progenitors were first cultured in neurobasal medium with B27 supplement for about 24 hours, then the medium was replaced with neurobasal medium containing N2 supplement and different concentrations of IL3 were added. The cultures were maintained for 3 days and cell viability was determined. IL3 has significant effects on this culture condition on progenitors (**d**) and neurons (**e**) (n = 8 for control group and 20 ng/ml group, n = 16 for other groups). *y*-axis, cell viability (normalized to control), *x*-axis, concentration of IL3 (ng/ml). Data are expressed as mean ± s.e.m. *P<0.05, **P<0.01 (two-tailed Student’s *t-test*). (**f**) IL3 activates PI3K-AKT, MAPK1/2 and Gsk3β signal pathways in MEG01 cell line. (**g**) IL3 activate MAPK, JAK/STAT, and PI3K/AKT pathways in primary cultured neural progenitors. The phosphorylation level of AKT, MAPK1/2, JAK2 and GSK3β was increased after IL3 treatment.

IL3 is known to activate three signaling pathways, the JAK/STAT, the MAPK, and the PI3K/AKT pathways, among which the MAPK signal pathway regulates cell proliferation [40]. Since we found IL3RA co-expressed with the cell proliferation markers Ki67 and pH3 (**Fig. S14**), IL3 could play a role in promoting the proliferation of neural progenitors. We thus examined the effect of IL3 treatment on the proliferation of neural progenitors isolated from E13.5 cortex. We first verified the expression of IL3RA and IL3RB in cultured neural progenitors (**Fig. S15a**). Anti-pH3 immunolabeling showed that the number of proliferating cells was significantly increased in IL3-treated samples compared to the controls (**Fig. 4a,b**), which is consistent with published observations [41]. Western blotting confirmed that IL3 could activate MAPK pathway in both MEG01 cells (**Fig. 4f**) and cultured neural progenitors (**Fig. 4g**). The phosphorylation of MAPK1/2 was significantly increased after IL3 treatment, indicating that IL3 can activate proliferation pathway in neural progenitors. We also investigated another proliferation related pathway, the JAK/STAT pathway. We found that JAK2 phosphorylation was increased after IL3 treatment (**Fig. 4g**), further supporting the involvement of IL3 in the proliferation of neural progenitors.

We next tested whether IL3 could drive neuronal differentiation *in vitro*. The progenitor cells were cultured under differentiation condition, treated with IL3, and the expression level of cell type-specific markers was measured by quantitative PCR. We found that IL3 had no effect on neuronal differentiation *in vitro*. After IL3 (10 ng/ml) treatment, the expression of all tested genes was not changed significantly (**Fig. S16**). Collectively, these data suggest that IL3 promotes the proliferation of neural progenitors through the activation of MAPK and JAK/STAT pathways, but has no effects on neural differentiation.

### Neurotrophic Effects of IL3 on Neural Progenitors and Neurons

IL3 is reported to have trophic effects on neurons [42]. It promotes the survival of sensory neurons and protects against neuronal death induced by FeSO4 and Aβ [43], [44]. We speculated that IL3 might also have similar trophic effects on cortical neural progenitors. To test this, we determined cell viability of cultured neural progenitors and neurons in media with different growth factors and supplements. The results suggest that when the nutrition is deficient, the IL3 pathway could be activated to protect against cell death induced by starvation (**Fig. S15b,c**, **Fig. 4c,d**). Similar results were obtained on neurons when cultured using the previously reported culture method [43] (**Fig. 4e**).

The Bcl-x_L_ was reported has a role in neuronal survival mediated by IL3 pathway [43], so we studied the expression of Bcl-x_L_ in neural progenitors and found there was no significant change after treated with IL3 (**Fig. S15e**). The signaling through the PI3K/AKT pathway is one of the most potent intracellular mechanisms to promote cell survival. It is well established that IL3 can activate the PI3K/AKT pathway [40]. To further study the mechanism of neural progenitor survival mediated by IL3 and to test if the PI3K/AKT pathway participates in IL3-mediated survival of neural progenitors, we studied the interactions between IL3 and AKT1 in neural progenitors by western blotting. In untreated progenitors, the level of phosphorylated AKT (the active form) is low (**Fig. 4g**). However, the level of phosphorylated AKT was dramatically increased after IL3 treatment (**Fig. 4g**). In addition, analysis of three AKT1 SNPs indicated significant association with brain volume (**Table S5**). Taken together, these results indicate that IL3 promotes the survival of neural progenitors by activating the PI3K/AKT pathway.

### IL3 Activates Estrogen Receptor α and β *in vitro*

As shown above, the genetic association of the IL3 SNPs with brain volume was female-specific in Chinese. To test whether estrogen could regulate the expression of IL3, we treated the K562 cell line (which expresses both IL3 and estrogen receptor (**Fig. S17a**)) with estrogen and found no overt change of IL3 expression (**Fig. S17c**–**e**), while the expression of TFF1 as a control was significantly increased after estrogen treatment (P<0.001) (**Fig. S17b**–**d**). These results suggest that the transcription of IL3 could not be directly regulated by estrogen.

**Figure 5:**
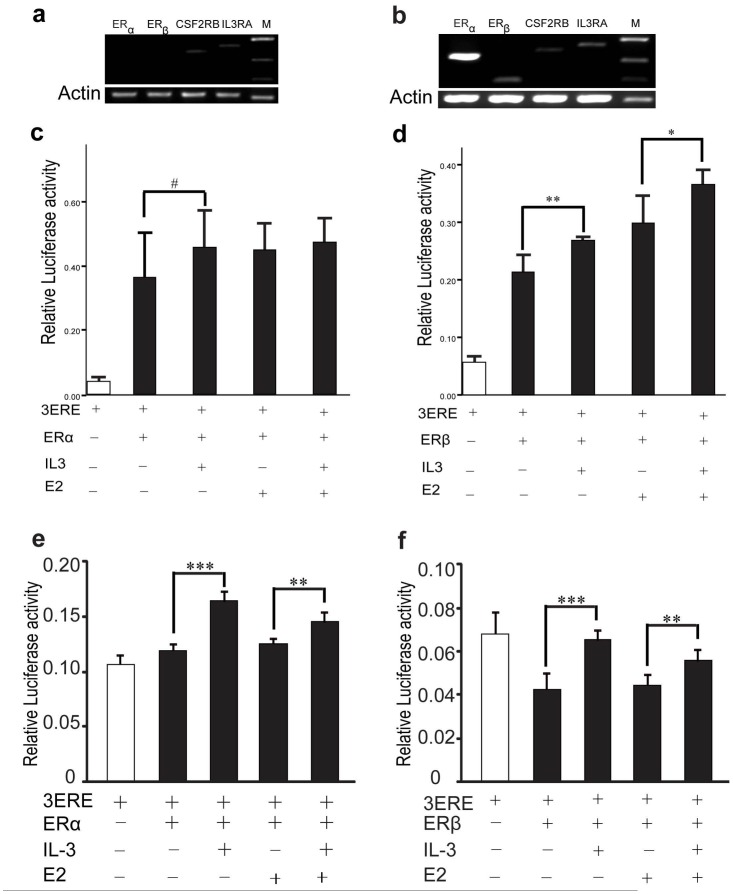
IL-3 activates estrogen receptors in MEG01 and HEK293T cells. There are two estrogen receptors, ER_α_ and ER_β_. MEG01 and HEK293T cell lines were used to test whether IL3 can activate estrogen receptors. Expression of IL-3 receptors (IL3RA, CSF2RB) were verified by RT-PCR in MEG01(a) and HEK293T (b) cell lines. In HEK293T cell line, we also detected the expression of estrogen receptors (ER_α_ and ER_β_) (b). Constructs containing three repeats of estrogen response element (3ERE) and estrogen receptor (ER_α_ or ER_β_) were co-transfected into MEG01 and HEK293T cell lines prior to IL-3 or estrogen (E2) treatment. IL-3 can activate ER_α_ and ER_β_ in both cell lines (c-d for MEG01 and e-f for HEK293T), and this effect was enhanced by the estrogen, indicating there were interactions between IL-3 and estrogen activation. Data are expressed as mean ± s.e.m. (three independent assays, each containing 3 replicates). ^#^P<0.10, *P<0.05, **P<0.01, ***P<0.001.

**Figure 6:**
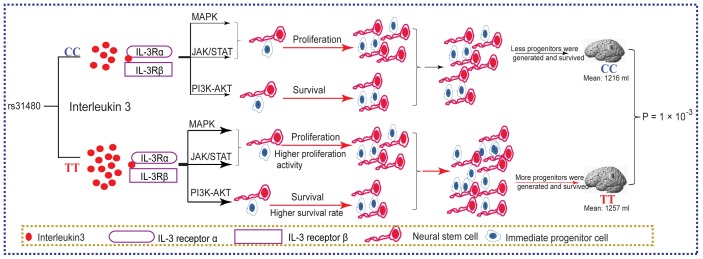
Model for regulation of brain volume by IL3 genetic variation and expression level. Individuals with different genotypes at rs31480 have differential expression level of IL3 (individuals with TT genotype have higher IL-3 expression than CC carriers), which lead to differential activation of signaling pathways mediated by IL3. The differential activation of signaling pathways further influence the proliferation and survival of neural progenitors, eventually lead to brain volume variation for individuals with different genotypes at rs31480.

It was reported that the MAPK and PI3K/AKT pathways could activate the estrogen receptor (ER) [45], [46]. To investigate whether IL3 could activate ER genes through the two regulated pathways, we constructed three vectors, 3ERE-PGL3 (contains three repeats of estrogen response element (ERE)), ER_α_ and ER_β_ respectively. We first studied the expression of IL3 receptors and estrogen receptors in MEG-01 and HEK293T cell lines and we found IL3 receptors were expressed in both cell lines (**Fig. 5a,b**). The vectors (3ERE-PGL3 and ER_α,_ or 3ERE-PGL3 and ER_β_) were then co-transfected into the IL3 receptor-expressing MEG-01 cell line, followed by IL3 or E2 treatments. As expected, IL3 could activate both ER genes, especially ER_β_ (**Fig. 5c,d**). In addition, the activation of ER_β_ induced by E2 was enhanced by IL3. We confirmed these results in HEK293T cells (**Fig. 5e,f**). Hence, the activation of ER genes by IL3 and estrogen and the interaction between them may explain the sex-specific functional effects of the sequence polymorphism at rs31480 on brain volume in females. The reported association studies on SCZ patients is consistent with our observation, in which IL3 showed a significant association with schizophrenia only in females. The hypothesized sex-specific regulation of brain volume is illustrated in **Fig. 6** **and Fig. S18.**

## Discussion

Brain volume is an important quantitative trait that underlies our most complex cognitive abilities and human evolution is characterized by a dramatic expansion in brain size and complexity. Undoubtedly, our unique genetic makeup played a decisive role in our enlarged brain and human brain size must be highly regulated during development. The hypothesis that SCZ is a brain disease unique to humans [47] suggests that SCZ susceptibility genes may regulate the unique features of human brain development and dysfunction of these genes may disrupt the normal development of the human brain. In this study, we provide evidence that IL3 may regulate human brain volume variation. First, our genetic association results strongly suggest the association between IL3 and brain volume. IL3 is located in 5q31.1, one of the most successfully replicated regions that may harbor SCZ susceptibility genes [26]–[35]. In fact, 5q23–31 was ranked number 2 of all chromosomal regions implicated to harbor SCZ susceptibility genes in a genome-wide meta-analysis of SCZ [48]. As accumulating data support that SCZ is a neurodevelopmental disorder, it’s likely that there are potential genes in 5q23.2–33.1 that regulate brain development. However, though studies repeatedly found association between genes located in 5q23.2–33.1 and SCZ, the detailed expression pattern of these genes and their function during brain development is not known. Here, for the first time, we detailed the spatiotemporal expression pattern of IL3 and its receptors in developing mouse brain and we found IL3RA is mainly expressed in neural progenitors and neurons, which also support the importance of IL3 signaling pathway in brain development. Also, our *in vitro* proliferation and survival assays further validate the pivotal roles of IL3 in the development of central nervous system. Collectively, these results provide novel insights to the involvement of IL3 in brain development, supporting the neurodevelopmental hypothesis of schizophrenia.

It should be noted that during the initial screening in the Chinese sample, we used cranial volume as proxy of brain volume. Though cranial volume is not exactly equal to brain volume, the correlation between these two variables is very high [37]. More importantly, we have successfully identified the association between cranial volume and MCPH1 gene by applying this method in our previous study [9]. In addition, the successful replication of our initial findings in genetically divergent populations further support the reliability of our method.

We realized that the association data and the functional data did not refer to the same SNP, which could be explained by several possible reasons. First, though rs3916441 has the smallest p value in our screening sample, it is located about 27 kb upstream of IL3, and the likelihood of rs3916441’s direct regulation of IL3 expression is relatively small. Second, the p value of rs3916441 and rs31480 is very close in our screening sample, and they are highly linked (r^2^ = 0.89) in Chinese. Hence, the functional data suggests rs31480 is probably the causal SNP in brain volume regulation. Nevertheless, we have successfully replicated the significant associations of IL3 variants with brain volume in BIG sample (rs3916441), and we also observed a marginal significant association in CBDB/NIMH sample (rs3916441). Although the association of these SNPs did not reach genome-wide significance, considering the non-overlap of the studied samples and different genetic backgrounds of Chinese and Europeans, IL3 is likely an authentic gene contributing to brain volume variation in general populations. To date, no genes have been shown significantly associated with brain volume and only a few genes were associated with intracranial volume in recent genome-wide association studies [11]–[13], suggesting an extremely complicated genetic regulation of brain volume.

Growing evidence have suggested that the interaction between immune and nervous systems may play an important role in the pathogenesis of schizophrenia [49]. The immune and nervous systems interact with each other through cytokines, a family of proteins that are secreted by a specific group of cells of the immune system and have pleiotropic effects on many cell types, including proliferation, differentiation, and survival. IL-3 is a cytokine that induces growth and differentiation of hematopoietic stem cells and a variety of cell types originating in the bone marrow. Recent studies have demonstrated the important role of IL3 in the central nervous system (CNS). It is expressed in the hippocampus and cortices of normal mouse brain [50], and it stimulates the growth and proliferation of microglial cells [51], [52]. Studies also found that IL3 facilitates the survival of sensory neurons significantly and stimulates the formation of the neural network [53]. In addition, IL-3 has been found to be able to promote the process extension of cultured cholinergic [42] and prevent delayed neuronal death in the hippocampus [43]. In fact, rat interleukin 3 receptor β-subunit was cloned from cultured microglia [54], and disruption of IL3 production in brain led to neurologic dysfunction [55]. All of these studies strongly suggest that IL3 is a pivotal protective factor for CNS. More importantly, Chen *et al.* recently reported that IL3 was significantly associated with brain disease such as schizophrenia in three independent Irish samples [34], [56], [57]. IL3 receptors, including IL3RA and CSF2RB (or IL3RB), were also found significantly associated with schizophrenia in three different populations [58]–[60]. In addition, decreased IL-3 levels in the first-episode and drug-naïve patients with schizophrenia was also reported [61]. These convergent evidences strongly indicate the involvement of the IL3 pathway in schizophrenia. Interestingly, we noticed that rs3916441, which is most significantly associated with brain volume, was also significantly associated with schizophrenia in females [34], implying the interaction between IL3 and gender may play vital roles in normal brain development and schizophrenia susceptibility. Though many investigations support the involvement of IL3 in brain function and schizophrenia, the precise expression pattern of IL3 and its receptors in developing brain is not well characterized and it’s not clear how genetic variation within IL3 affect brain development and schizophrenia susceptibility.

In summary, we have demonstrated that IL3 plays crucial roles in the development of the central nervous system. We identified a genetic variant (rs31480) in the promoter of IL3 that is significantly associated with brain volume in the general population. This polymorphism influences the expression of IL3 and the differential IL3 expression of the two alleles at rs31480 can influence neural progenitor pool expansion and maintenance during neurodevelopment. Furthermore, our findings that IL3 can promote proliferation and survival of neural progenitors further support the proposed novel role of IL3 in the central nervous systems.

## Materials and Methods

### Samples

Our screening samples are from Yunnan province of southwestern China (n = 1,013). Replication samples included samples from the Alzheimer’s Disease Neuroimaging Initiative (ADNI; n = 204), samples from the US National Institute of Health (CBDB/NIMH; n = 188) and samples from the Dutch Brain Imaging Genetics study (BIG; n = 486).

### Screening Samples: Chinese Samples

The detailed information of the sample screening was described in our previous study [9]. Briefly, a total of 1,013 unrelated healthy individuals including 460 males and 553 females were included. The identities of the subjects were self-declared and confirmed by their written ID profiles. All the sampled individuals are from Yunnan province of southwestern China. Written informed consents for this study were obtained from all the subjects, and the research protocol was approved by the internal review board of Kunming Institute of Zoology, Chinese Academy of Sciences. The ages of the 1,013 individuals range from 19 to 28 years with 98% of them being 21–26 years old.

### Replication Samples: ADNI Sample

The MRI and genotyping data in this replication sample were obtained from the Alzheimer’s Disease Neuroimaging Initiative (ADNI) database (adni.loni.ucla.edu). One goal of ADNI has been to test whether serial magnetic resonance imaging (MRI), positron emission tomography (PET), other biological markers, and clinical and neuropsychological assessment can be combined to measure the progression of mild cognitive impairment (MCI) and early Alzheimer’s disease (AD). For up-to-date information, see www.adni-info.org. The ADNI participants consist of patients with AD, patients with MCI, and elderly healthy individuals. They were aged 55–90 years and recruited from 59 sites across the U.S. and Canada. Written informed consent was obtained from all 822 participants and the study was conducted with prior Institutional Review Board approval. Of 822 participants, 204 unrelated non-Hispanic Caucasian healthy controls were used in this study [62].

### Replication Samples: CBDB/NIMH Sample

All subjects are the healthy control participants of Clinical Brain Disorders Branch/National Institute of Mental Health Sibling Study, a study aimed at identifying schizophrenia susceptibility genes and related intermediate biologic phenotypes [63]. Subjects with good quality of structural data and genotyping were included the study.

### Replication Samples: BIG Sample

In this study, a total of 486 healthy control subjects aged 18–35 years from the Brain Imaging Genetics (BIG) study at the Donders Institute for Brain, Cognition and Behaviour of the Radboud University Nijmegen (Medical Centre) were included. The BIG study is a study of self-reported healthy individuals included into earlier imaging studies at the Donders Centre for Cognitive Neuroimaging. Subjects are of European Caucasian descent and generally highly educated [64]. The study was approved by the regional medical ethics committee (CMO regio Arnhem/Nijmegen) and all participants provided written informed consent prior to participation.

### Measurement of Cranial Volume: Chinese Screening Sample

The cranial volume was measured and calculated as described in our previous study [9]. Three principal dimensions of the cranium were measured including 1) Maximum antero-posterior length (L, measured between glabella and the inion). 2) Maximum breadth (B, biparietal diameter; measured between two parietal eminences). 3) Cranial height (H, basi-bregmatic height, measured between the internal acoustic meatus to the highest point of the vertex). Then the cranial volumes were computed using the following formula [9], [65]: Male, 0.337 (L-1.1) (B-1.1) (H-1.1) +406.01 cc; Female, 0.400 (L-1.1) (B-1.1) (H-1.1) +206.60 cc.

### Measurement of Brain Volume: Replication Samples (ANDI)

3D T1-weighted brain MRI scans were acquired using a sagittal 3D MP-RAGE sequence following the ADNI MRI protocol [66]. Baseline 1.5T MRI scans from 204 participants were downloaded from the ADNI public website (http://www.loni.ucla.edu/ADNI/) onto local servers at Indiana University School of Medicine. As detailed in previous studies [67], FreeSurfer V4 software (http://surfer.nmr.mgh.harvard.edu/), a widely employed brain segmentation and cortical parcellation tool, was used to label cortical and subcortical tissue classes using an atlas-based Bayesian segmentation procedure and to extract the measure of brain volume.

### Measurement of Brain Volume: Replication Samples (CBDB/NIMH)

All structural MRI were acquired on a 1.5 Tesla GE scanner (GE Medical Systems, Milwaukee, Wisconsin) using a T1-weighted spoiled gradient recalled (SPGR) sequence (repetition time, 24 ms; echo time, 5 ms; number of excitations, 1; flip angle, 45°; matrix size 256×256; FOV 24×24 cm^2^), with 124 sagittal slices (0.94×0.94×1.5 mm^3^ resolution). Images were processed using the FreeSurfer [68] toolbox (version 5). Total Brain Volume (TBV) and Total Grey Matter volume (TGM) measurements were calculated as previously described [63], [69]. TGM was defined as sum of tissue probabilities for the grey matter region. TBV was defined as the sum of total gray matter volume, total white matter volume, and cerebrospinal fluid.

### Measurement of Brain Volume: Replication Samples (BIG Sample)

Subjects were scanned at 3 Tesla (n = 486) MRI scanners and T1-weighted structural magnetic resonance imaging data (3D MPRAGE) were acquired (more information on the image acquisition can be found in our previous study [70]). All scans covered the entire brain and had a voxel-size of 1×1×1 mm^3^. To calculate total brain volume, raw DICOM MR imaging data were converted to NIFTI format using the conversion as implemented in SPM5 (http://www.fil.ion.ucl.ac.uk/spm/software/spm5/). Normalizing, bias-correcting, and segmenting into gray matter, white matter, and cerebrospinal fluid was performed using the VBM toolbox (VBM5.1 Toolbox version 1.19) in SPM using priors (default settings). This method uses an optimized VBM Protocol [71], [72] as well as a model based on Hidden Markov Random Fields (HMRF) developed to increase signal-to-noise ratio [73]. Total volume of gray matter, white matter, and cerebrospinal fluid was calculated by adding the resulting tissue probabilities. Total brain volume was defined as the sum of white matter and gray matter volume.

### SNP Selection, SNP Tagging, Genotyping and Sequencing

SNP selection was based on the previous association studies including our recent data [33]–[35]. We focused on the four genes that identified by Chen *et al.* recently using systematically mapping in large independent samples. In addition, our recent data and LD in Chinese were also considered. We selected 8 SNPs for the initial screening (rs3756295, rs40396, rs1291602, rs31251, rs1355095, rs2240525, rs3914025, rs31400), additional SNPs were included according to the association significance and whether they are tagging SNPs. Totally, we selected 20 SNPs for fine mapping. The 20 SNPs were genotyped using the SNaPshot method (Applied Biosystems). We sequenced the IL3 gene (including the 500 bp upstream and downstream, respectively) in 150 randomly selected individuals through direct sequencing. The conservation analyses were performed by using UCSC genome browser [74]. (http://genome.ucsc.edu/).

### Prediction of DNA-binding Motifs

We used Dragon ERE finder [75], a web-based program for identification and interactive analyses of estrogen response elements (EREs) to predict EREs in the upstream region of IL3. AliBaba (http://www.gene-regulation.com/pub/programs/alibaba2/index.html) was used to predict and compare DNA-binding motifs in the promoter region with alternative alleles.

### Cell Culture, Treatment, and RNA Extraction

K562 cells were routinely cultured in DMEM (Gibco) supplemented with 10% FBS (Hyclone), 100 u/ml penicillin and 100 ug/ml streptomycin. Before treatments, the cells were maintained in phenol red-free DMEM containing 10% dextran-coated charcoal-stripped *fetal bovine serum* (*DCC*-*FBS*) (Hyclone) for a minimum of 3 days with the media changed every day. Cells were treated with 10 nM 17-beta-estradiol (E2) (Sigma) for 2 to 24 hours. Total RNA was harvested and prepared using TRIzol (Invitrogen) following the manufacturer’s instructions.

### Quantitative Real-time PCR

Reverse transcription PCR (RT-PCR) was performed using the Omniscript RT Kit (Qiagen) following the manufacturer’s instructions. We carried out real-time quantitative PCR using gene specific primers, and the fold change in expression was calculated using the ΔΔC_t_ (threshold cycle) method. The GAPDH was used as the internal control.

### EMSA

EMSAs were performed with a Lightshift™ chemilumescent EMSA kit (Pierce). The single-strand oligonucleotides were biotinylated with Biotin 3′ End DNA labeling Kit (Pierce) and then annealed to form double strands. The nuclear extracts of MCF-7 and U2OS were prepared by CelLytic™ NuCLEAR™ Extraction kit (Sigma). HeLa nuclear extracts were purchased from Santa Cruz Biotech. The binding reactions were performed for 20 mins at room temperature in 10 mM Tris-HCl (PH 7.5), 1 mM MgCl_2_, 0.5 mM EDTA, 0.5 mM DTT, 50 mM NaCl, 50 ug/ml poly (dI-dC)(dI-dC) and 4% glycerol, 35 fmol biotin 3′-end -labeled double-stranded oligonucleotides, and purified recombinant SP1 protein (Alexis) or nuclear extracts. After incubation, samples were separated on a native 6% polyacrylamide gel and then transferred to a nylon membrane. The positions of biotin end-labeled oligonucleotides were detected by a chemilumescent reaction with streptavidin-horseradish peroxidase according to the manufacturer’s instructions and visualized by autoradiography. For competition assays, we pre-incubated 100-fold excess of unlabeled oligonucleotide probe with SP1 or nuclear extracts before adding the biotin-labeled probe. The nucleotide sequences of the double-stranded oligonucledtides with either T or C allele are:

T-allele: 5′-TGCACATATAAGGCGGGAGGTTGTTGCCAACGCTTCAGAGC-3′.

C-allele: 5′-TGCACATATAAGGCGGGAGGCTGTTGCCAACGCTTCAGAGC-3′.

### Promoter Cloning and Reporter Gene Assays

To construct IL3 promoter, we amplified fragments encompassing nucleotides -436 to +164 (relative to the ATG start codon at +1) of IL3 by PCR from genomic DNA of two individuals homozygous with respect to the corresponding genotypes (TT and CC) for rs31480, using primers tailed with *Xhol* and *HindIII* restriction sites, and directionally subcloned them into the *Xhol* and *HindIII* sites of the pGL3-Basic expression vector (Promega). We verified all recombinant clones by bi-directional DNA sequencing. HeLa, COS-7, CHO, and SK cells were routinely cultured in DMEM supplemented with 10% FBS with antibiotics. The cells were plated at 2.5×10^5^ cells per well in a 24-well plate the day before transfection and incubated overnight at 37°C in 5% CO_2_. Transient transfection assays were conducted in these cells using the Lipofectamine 2000 transfection reagent (Invitrogen), all assays were performed in at least three independent experiments with minimum of three replicates. The reporters containing either T allele or C allele were transfected into these cells together with a *Renilla* luciferase control vector. After 24h incubation, we collected the cells and measured luciferase activity using the Dual-Luciferase Reporter Assay System (Promega).

### 3ERE Cloning and IL3 Activation Assays

Three repeats of Estrogen Response Elements (EREs) tailed with *Xhol* and *HindIII* restriction sites were synthesized and annealed to form double-stranded nucleotides. The sequence is 5′-CCG CTCGAG TA GGTCA GCG TGACC TA TA GGTCA GCG TGACC TA TA GGTCA GCG TGACC TA AAGCTT GGG-3′. We directionally cloned it into the pGL3-Basic vector after restriction enzyme digestion. We confirmed the construct by sequencing. The estrogen receptor alpha and beta vectors were kindly provided by professor Sylvie Mader (Faculté de Médecine, Université de Montréal) and Leigh C. Murphy (University of Manitoba). MEG-01 and HEK293T, IL3 receptor expression positive cell lines were cultured in PRMI 1640 and DMEM respectively supplemented with 10% FBS, 2 mM L-glutamine, 1 mM Sodium pyruvate and 1% antibiotics. Co-transfection of 3ERE-pGL3 (2 ug), ER_α_ (1 ug) or ER_β_ (1 ug) were performed by using a Nucleofector Device from Amaxa Biosystems (Lonza Cologne AG, Germany) in MEG01 cell line, while co-transfection in HEK293T cell line were used Lipofectamine 2000 transfection reagent (Invitrogen) as previously described, and with pRL-TK as the internal control. After six hours incubation, 17-beta-estradiol and recombinant human IL3 protein (Invitrogen) were added into the medium with a final concentration of 10 nM. Cells were harvested and their luciferase activity was measured after additional 24 h incubation. All assays were performed in at least three independent experiments with a minimum of triplications.

### Statistical Analysis

Hardy-Weinberg equilibrium of each SNP was assessed by using GENEPOP (v 4.0) [76]. Association of single SNP with total brain volume (TBV) and their additive effects on this quantitative trait were tested by utilizing PLINK or SAS statistical software using the linear regression option, with age, sex and IQ (optional) as covariates; for the analyses on total gray/white matter, TBV was also considered as a covariate [77]. To account for sex-specific effects, we used a statistical model where the mean effect of SNP dose on the phenotype was allowed to differ for the two sexes. The p-value was adjusted by the conservative Bonferroni correction according to the number of independent SNPs and the divided internal samples separated by sex. We used the Haplo Stats [78] to infer the haplotype frequency and to perform the haplotype association test. We used the Haploview [79] to calculate pairwise LD indices r^2^ and D’, to define LD blocks and to select the tag SNPs. Haplotypes were inferred with the PHASE program by the Bayesian statistical methods based on the genotype data [80]. Sequence alignment and assembly were conducted by DNASTAR software package. The analysis of quantitative PCR data was based on the ΔC_t_ values.

### Immunohistochemistry

The C57BL/6J mice were used in this paper and all animal procedures described herein were approved by the University Committee of Animal Resources at the University of Rochester. For the purposes of staging embryos, noon of the day a vaginal plug was detected was taken to be embryonic day 0.5 (E0.5). Mice were deeply anesthetized, perfused transcardially with PBS, followed by 4% PFA in PBS, pH 7.3. Then the brains were dissected, postfixed in 4% PFA in PBS at 4°C overnight, washed three times in PBS, and cryoprotected in 30% sucrose in PBS before rapid freezing in OCT compound (TissueTek). For antigen retrieval, cryosections (20 µm) were heated in 10 mM citrate buffer (pH 6.0) at 95°C for 10 min. The sections were permeabilized and blocked in PBS plus 0.1% Tween-20, 5% horse serum, and incubated with primary antibody overnight at 4°C, washed in PBS three times and incubated with fluorescently labeled secondary antibody for 1 h at room temperature. The primary antibodies used were mouse monoclonal anti-IL3RA (Santa Cruz, 1∶100), rabbit polyclonal anti-IL3RA (Santa Cruz, 1∶100), rabbit polyclonal anti-IL3RB (Santa Cruz, 1∶100), goat polyclonal anti-IL3 (Santa Cruz, 1∶100), rabbit monoclonal anti-β-III tubulin (Tuj1) (Covance, 1∶1000), mouse anti-NeuN (Chemicon, 1∶300), goat anti-SOX2 (Santa Cruz, 1∶500), rabbit anti-Nestin (Abcam, 1∶300), rabbit anti-TBR2 (Millipore, 1∶200), rabbit anti-pH3 (Santa Cruz, 1∶200), rabbit anti-PROX1 (Covance, 1∶1000), anti-estrogen receptor β (Santa Cruz, 1∶100), rabbit anti-Ki67 (Novocastra, 1∶1000), rabbit anti-GFAP (1∶2000). Secondary antibodies used were Alexa Fluor 488 (Invitrogen, 1∶500), 546 (Invitrogen, 1∶500) conjugated to donkey anti-mouse, rabbit or anti-goat (Invitrogen). DNA was stained with 4′,6-diamidino-2-phenylindole (DAPI; Molecular Probes). Images were acquired with a Zeiss laser confocal microscope and analysed with LSM 510 software (Carl Zeiss).

### Primary Cultures

For neural progenitor’s culture, cerebral cortices from C57BL/6 mice embryos (E12.5–14.5) were dissected in HBSS solution (Invitorgen), the meninges and other parts were removed under dissecting microscope and only the cortices were retained. After several washes with HBSS, the cortices were minced and dissociated mechanically with trituration, filtered through a 70 µm cell strainer (BD Falcon). Then, the cells were culture in neurobasal medium (Invitrogen) or DMEM/F12 (Millipore) with different supplements and growth factors according to experimental requirements. For neurons culture, the procedures are same with neural progenitors except the age of mice embryos (E14.5–17.5).

### Proliferation Assays

For generation of neurospheres, the dissociated cells from E14.5–17.5 mice cortices were cultured for 2–5d in the medium containing neurobasal medium, B27 (Invotrogen) and N2 (Invitrogen) supplements, 100 U/mL penicillin, 100 µg/mL streptomycin, FGF2 (10 ng/ml), and EGF (10 ng/ml). For monolayer cultures, the generated neurospheres were collected and spun down (200 g for 5 minutes), then triturated with a Pasteur pipette to obtain single cells. The single cell suspensions were replated onto poly-L-lysine (50 µg/ml)/laminin (10 µg/ml)–coated Lab-Tek Chamber Slide (Thermo Scientific). Cells were treated with different concentrations of recombinant mouse IL3 (10 and 100 ng/ml) and cultured in above medium. Untreated cells served as control. After 24 or 48 hours culture, cells were fixed with 4% PFA and subjected to mmunohistochemistry. Immunostaining and morphometry were carried out to assess the numbers of proliferating progenitors (Ki67 and pH3 positive). More than 4 random microscopic fields (20×) were analyzed and about 8000 cells were counted for each condition.

### Assessment of Differentiation Markers *in vitro*

To investigate whether IL3 can drive neural differentiation, we used quantitative real-time PCR method described previously [81] to evaluate the effects of IL3 on neural differentiation. First, neural progenitors were cultured in neurobasal medium (containing B27 supplements, N2 supplements,10 ng/ml FGF2 and 10 ng/ml EGF) for 2 days, then FGF2 and EGF was removed and replaced by neurobasal medium with 1% serum (vol/vol), B27 supplements, N2 supplements and IL3 (10, 100, and 200 ng/ml). Four days after addition of recombinant mouse IL3 (Invitrogen), cells were harvested for the RNA extraction. Untreated cells served as control. RNA was isolated using Trizol reagent (Invitrogen) according to the manufacturer’s instructions and treated with DNase I (Fermentas). cDNA was synthesized from 3 µg total RNA using oligo-dT primers and Superscript III Reverse Transcriptase (Invitrogen). Quantitative PCR was performed by using the Bio-Rad iCycler & iQ Real-Time PCR Systems. The following primer pairs were used: mouse Sox2-F, GCGGAGTGGAAACTTTTGTCC, mouse Sox2-R, CGGGAAGCGTGTACTTATCCTT; mouse Nestin-F, CCCCTTGCCTAATACCCTTGA, mouse Nestin-R, GCCTCAGACATAGGTGGGATG; mouse β-III-tub-F, TAGACCCCAGCGGCAACTAT, mouse β-III-tub-R, GTTCCAGGTTCCAAGTCCACC; mouse GFAP-F: CCCTGGCTCGTGTGGATTT, mouse GFAP-R: GACCGATACCACTCCTCTGTC; mouse ENO2-F: GTCCCTGGCCGTGTGTAAG, mouse ENO2-R: CATCCCGAAAGCTCTCAGC; mouse nestin-F: CCCTGAAGTCGAGGAGCTG, mouse nestin-R: CTGCTGCACCTCTAAGCGA; mouse PLP1-F: TGAGCGCAACGGTAACAGG, mouse PLP1-R: CCCACAAACTTGTCGGGATG. The iCycler PCR analysis was performed using the SYBR Green master mix, according to the manufacturer’s recommendations (BioRad). The specificity of product was ensured by melting curve analysis and agrose gel electrophoresis. cDNA content of samples was normalized to the expression of GAPDH.

### Cell Viability Assays

To investigate whether IL3 has protective or trophic effects on neural progenitor’s survival, cerebral cortices from E12.5 or E13.5 mice embryos were dissociated and the isolated cells (5×10^4^) were plated onto poly-L-lysine coated 96-well plate (Corning). The survival of neural progenitors was investigated by three culture conditions. Firstly, we studied the neurotrophic effects of IL3 on neural progenitors through culturing the progenitors in the absence of any factor (with neurobasal medium only) and in the presence of IL3 (0.02–50 ng/ml). Recombinant mouse IL3 was added into the medium after the culture initiated two hours. After 36 hours incubation, cell viability was determined by measurement of cellular ATP levels (CellTiter-Glo Luminescent Cell Viability Assay, Promega). Secondly, neural progenitors were first cultured in neurobasal medium supplemented with B27 and Glutamax (Invitrogen). On day 2 of culture, the medium was replaced with serum-free neurobasal medium containing N2 supplement and different concentrations of mouse IL3 (0.1–20 ng/ml). The cultures were maintained for 3 days and cell viability was measured. Thirdly, neural progenitors were first cultured in neurobasal medium supplemented with B27 and Glutamax. On day 2 of culture, IL3 was added into the medium and the cultures were maintained for 3 days and cell viability was measured. For studying the effects of IL3 on neurons, cerebral cortices from E16.5–E18.5 mice embryos were dissociated and cultured in DMEM/F12 medium with 5% FBS. On day 2 of culture, the medium was replaced with serum-free DMEM/F12 containing N2 supplement and different concentrations of IL3 (0.1–20 ng/ml). The cultures were maintained for 2 or 3 days and cell viability was determined as described above.

### Western Blotting

Neural progenitors from E13.5 mice were first cultured in neurobasal medium under proliferating condition (containing B27 supplement, Glutamax, 10 ng/ml FGF2 and EGF) for one week. To exclude the interference of other factors, the supplements, FGF2 and EGF were removed from the medium for about 16 hours prior to IL3 treatment (3 ng/ml). Proteins from MEG01 cells (IL3 treated) and neural progenitors were homogenized in RIPA lyses buffer (Cell signaling) containing a cocktail of protease inhibitor (Sigma Chemical, MO, USA) and phosphatase inhibitor (Cell Signaling). Proteins were quantified by BCA method (Pierce). Extracted protein (40 µg) was separated by SDS-polyacrylamide gel electrophoresis and transferred to PVDF or Nitrocellulose membrane by electrophoretic transfer. The membrane was blocked, incubated with primary antibodies for overnight at 4°C, washed three times with TBST, and then incubated with secondary antibody for 1 hour at room temperature. Antibodies used in western blot are as follows: Rabbit anti-phospho-AKT (Thr308) (Cell Signaling), Rabbit anti-AKT1 (Cell Signaling), Rabbit anti-phospho-JAK2 (Tyr 1007/1008) (Cell Signaling), Rabbit anti-JAK2 (Cell Signaling), Rabbit anti-phospho-GSK3β (Ser9) (Cell Signaling), Rabbit anti-phospho-ERK1/2 (Cell Signaling), Rabbit anti-ERK1/2 (Cell Signaling), Rabbit anti-GSK3β (BD), and Rabbit anti-actin (Abcam). Immunoreactivity was detected with an enhanced chemiluminescence system (Pierce, IL, USA) with colored markers (Fermentas) as molecular size standards.

## Supporting Information

Figure S1 **Linkage disequilibrium (LD) pattern of the studies SNPs in Chinese (CHB) and Europeans (CEU).** (a) In screening sample (CHB), they are four haplotype blocks and all the 7 highly significant association SNPs are located in block 2. (b) In CEU, they are five haplotype blocks and the 7 highly linked SNPs in CHB are disrupted. LD values (r^2^) for each pair of markers were calculated by Haploview (v4.2). Haplotype blocks were defined according to the criteria of Gabriel et al.
(PDF)

Figure S2 **The C allele of rs31480 is highly conserved in vertebrate.** rs31480 (box in red) is locates −16 bp upstream of the IL3 promoter, and only 10 bp downstream of the highly conserved TATA binding site (box in blue). The C allele (ancestral allele) is completely conserved in all of the listed species, implying functional importance of rs31480. Note that rs31480 is lies in a primates conserved region (up panel), also suggesting the importance of rs31480 in primates. TSS, transcription start site.
(PDF)

Figure S3 **Impacts of rs31480 on promoter activity in CHO, SK-N-SH and COS-7 cell lines.** Promoter activity of the construct with T allele is significantly higher than C allele in CHO (a) and COS-7 (b) cells. In SK-N-SH cells (c), the trend is same as CHO and COS-7 though the differences were not reached significant level. Values of relative luciferase activity are expressed as mean ± s.d. (results of a triplicate assay). ^#^P = 0.07, **P<0.005 (Student’s *t-test*).
(PDF)

Figure S4 **Expression of IL3RA in embryonic mouse brain (E12.5).** IL3RA is expressed in SOX2 positive cells in Frontal cortex and hippocampus, two regions that associated with higher level cognitive functions. Scale bar, 50 µm.
(PDF)

Figure S5 **IL3RA is mainly expressed in the neocortex region of mouse brain.** (a–f) Co-expression of IL3RA and IL3RB in agranular retrosplenial cortex (RSA), barrel field of the primary somatosensory cortex (S1BF). (g–l) Expression of IL3RA was also found in perirhinal cortex (Prh), primary Motor Cortex (M1) and secondary Motor Cortex (M2). Note that IL3RA positive cells were also expressed Tbr2 weakly, indicating they were not mature neurons. Scale bar, 25 µm.
(PDF)

Figure S6 **Expression of IL3RA and IL3RB in hippocampus.** (a–c) Co-expression of IL3RA and IL3RB in CA3 region of hippocampus. (d–i) IL3RA is expressed in CA1 and CA3 of hippocampus, some of IL3RA positive cells were Tuj1 positive, indicating they were mature neurons, whereas others were Tuj1 negative. Scale bar, 25 µm.
(PDF)

Figure S7 **IL3RA is expressed in hilus of dentate gyrus.** Prox1 was used to label the granule cell layer (GCL) of the dentate gyrus, note IL3RA positive cells in hilus were prox1 negative. Scale bar, 50 µm.
(PDF)

Figure S8 **Double immunostaining of IL3RA and SOX2 revealed co-localization of IL3RA and SOX2 in developing mouse brain.** (a–c) IL3RA expression cells were SOX2 positive in lateral septal nucleus, dorsal part (LSD), indicating they were neural progenitors. (d–i) In cortex, some IL3RA positive cells still express SOX2, but for many of IL3RA positive cells, expression of SOX2 was down-regulated or turned-off, demonstrating they were converted into immediate progenitors or neurons. Scale bar, 25 µm.
(PDF)

Figure S9 **Co-expression of IL3RA and IL3RB in mouse brain.** All IL3RA positive cells were also expression IL3RB. Scale bar, 25 µm.
(PDF)

Figure S10 **IL3RA is mainly expressed in neural progenitors at early embryonic stage.** (a–c) At E12.5, IL3RA is expressed in sox2 positive progenitors in frontal cortex. (b–e) Co-expression of IL3RA and nestin, a marker for neural progenitors. (f–h) At E14.5, IL3RA is expressed in some sox2 positive progenitors in cingulate cortex. Scale bar, 25 µm.
(PDF)

Figure S11 **Double immunostaining analysis of IL3RA and Tuj1 in the developing mouse brain.** At early stage of brain development (From E14.5-P1), IL3RA is not expressed in mature neurons (a–i). However, at P2 stage, a proportion of IL3RA positive cells are mature neurons as revealed by co-localization with tuj1 (j–l), a marker for mature neurons. Scale bar, 25 µm.
(PDF)

Figure S12 **IL3RA is expressed in immediate progenitor cells (IPCs).** IL3RA positive cells also express Tbr2 weakly, indicating these cells were immediate progenitors. Scale bar, 25 µm.
(PDF)

Figure S13 **IL3RB is expressed in neurons and glia cells.** (a–c) Co-immunofluorescence of IL3RB and NeuN revealed expression of IL3RB in mature neurons. (d–f) IL3RB also expressed in some glia cells (GFAP positive). Scale bar, 25 µm.
(PDF)

Figure S14 **Expression of IL3RA in proliferating neural progenitors.** Some IL3RA positive cells also expressed Ki67 and pH3, marker for proliferation cells, indicating they were active proliferation. Scale bar, 25 µm.
(PDF)

Figure S15 **Expression of IL3 receptors (IL3RA and IL3RB) in cultured neural progenitors and trophic effects of IL-3 on neural progenitors and neurons.** (a) IL3 receptors (IL3RA and IL3RB) were expressed in cultured neural progenitors (from E13.5 mice) as revealed by RT-PCR, however, βIL3 was not detected. (b) When there were 5% FBS in neurobasal medium, IL-3 has no trophic effects on neural progenitors. Neural progenitors from E12.5 mice were cultured in neurobasal medium (supplemented with 5% FBS and IL-3) for 36 hours then cell viability was measured. (c) IL-3 (1 ng/ml) significantly promotes survival of neurons when there were no any factors in neurobasal medium. Neurons from E17.5 mice were cultured in neurobasal medium (supplemented with B27 and Glutamax) for 12 days, then B27 and Glutamax were removed from medium and different concentrations of IL-3 were added. Cell viability was determined after IL-3 treatment for 2 days. (d) However, when there were B27 supplement in neurobasal medium, trophic effects of IL-3 on neurons was disappeared. Neurons from E18 mice were first cultured in neurobasal medium (supplemented with B27 and different concentrations of IL-3) for 24 hours, cell viability was then measured. **Y**-axis, cell viability (normalized to control); **X**-axis, concentration of IL-3 (ng/ml). Data are expressed as mean ± s.e.m. (n = 8 for each group). *P<0.05 (Student’s *t-test*). (e) The expression of BCL-xL was not regulated by IL3 in neural progenitors.
(PDF)

Figure S16 **IL3 has no effects on neural differentiation.** Neural progenitors were first cultured in neurobasal medium under proliferation condition (containing 10 ng/ml FGF2 and EGF), after 4 day’s culture, FGF2 and EGF were removed. Then 2% FBS and different concentrations of IL3 (10, 100, 200 ng/ml) were added. The cultures were maintained for 4 days and RNA was isolated for quantification. Relative gene expression was not changed for all of the tested genes, indicating IL3 has no effect on neural differentiation. Real-time PCR analysis of beta-III Tubulin (Tubb3) (a) and Enolase 2 (ENO2) (b), two neuron specific markers, GFAP (glia cells marker) (c), sox2 (neural stem cells marker) (d), Tbr2 (immediate progenitors marker) (e), and DCX (new born neurons marker) (f). None of these cell specific markers showed significant change after different concentration IL-3 treatment.
(PDF)

Figure S17 **IL-3 is not regulated by estrogen.** We first confirmed that K562 cell line expressed IL3 and estrogen receptors (ESR1 and ESR2) by RT-PCR (a). (b–c) Expression of TFF1 and IL3 was not changed after treated by vehicle (DMSO) for different times (0 h–24 h). TFF1, an estrogen response gene, showed significantly elevated after treated by estrogen (10 nM) (d), however, expression of IL-3 was not changed after estrogen treatment (e), indicating IL-3 is not regulated by estrogen. Data are expressed as mean ± s.e.m. (three independent assays, each containing 3 replicates). ^**^P<0.01.
(PDF)

Figure S18 **Model for sex-specific association of IL-3 and brain volume.** Different genotypes at rs31480 (TT vs. CC) influence IL-3′s expression in both males and females. However, since the estrogen receptors (ER) level is low in males, therefore, even the signaling pathways mediated by IL-3 were different in TT and CC carriers, the total activation level of ERs was not significant changed. But in females, the activation level of ERs was different between TT and CC carriers due to high level of ERs. In addition, estrogen can further enhance ER activity in females. As a result, signaling pathways mediated by ER were greatly activated in TT carriers than in CC carriers, which may influence brain development, eventually lead to difference of brain volume in TT and CC carriers at rs31480.
(PDF)

Table S1 **Marker characteristics and association significance in females.**
(DOC)

Table S2 **Core haplotype association analysis in females.**
(DOC)

Table S3 **Marker characteristics and association significance in males.**
(DOC)

Table S4 **Average cranial volumes of female individuals with three different genotypes at each of the seven SNPs covering IL-3.**
(DOC)

Table S5 **Marker characteristics of AKT1 and association significance.**
(DOC)
