# Supplementary material for: The Interleukin 3 Gene (IL3) Contributes to Human Brain Volume Variation by Regulating Proliferation and Survival of Neural Progenitors
Source: PLoS One. 2012 Nov 30;7(11):e50375. doi: 10.1371/journal.pone.0050375 (PMC3511536; doi:10.1371/journal.pone.0050375)

**Figure S1.** Linkage disequilibrium (LD) pattern of the studies SNPs in Chinese (CHB) and Europeans (CEU).

a

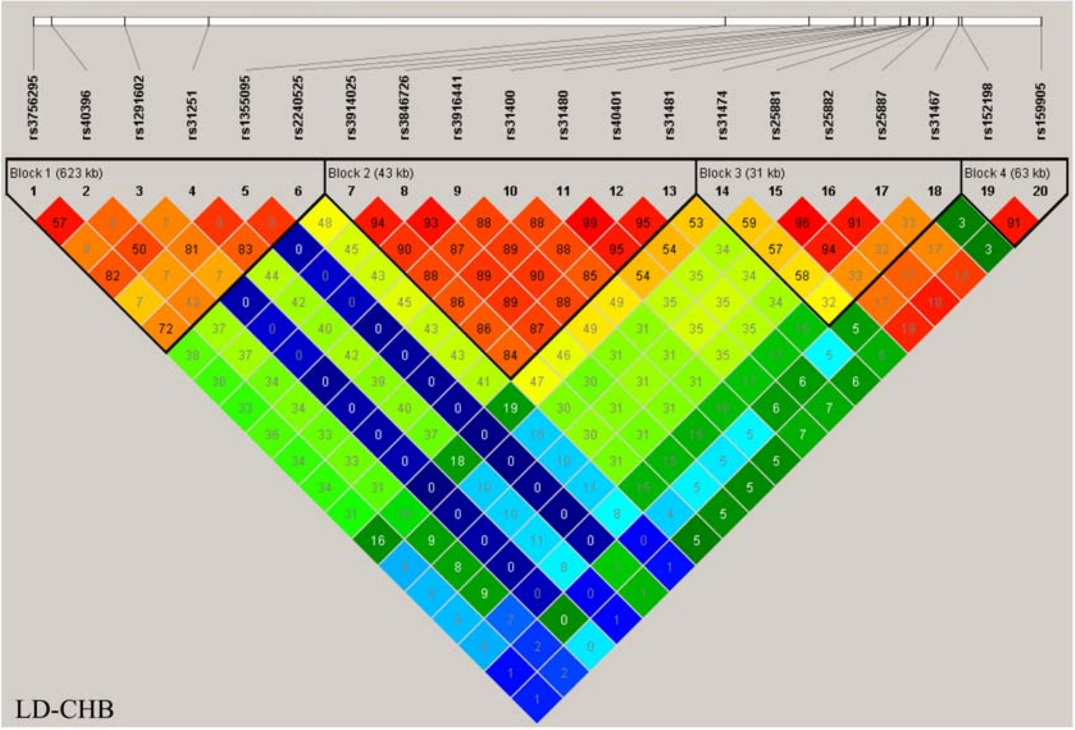


b

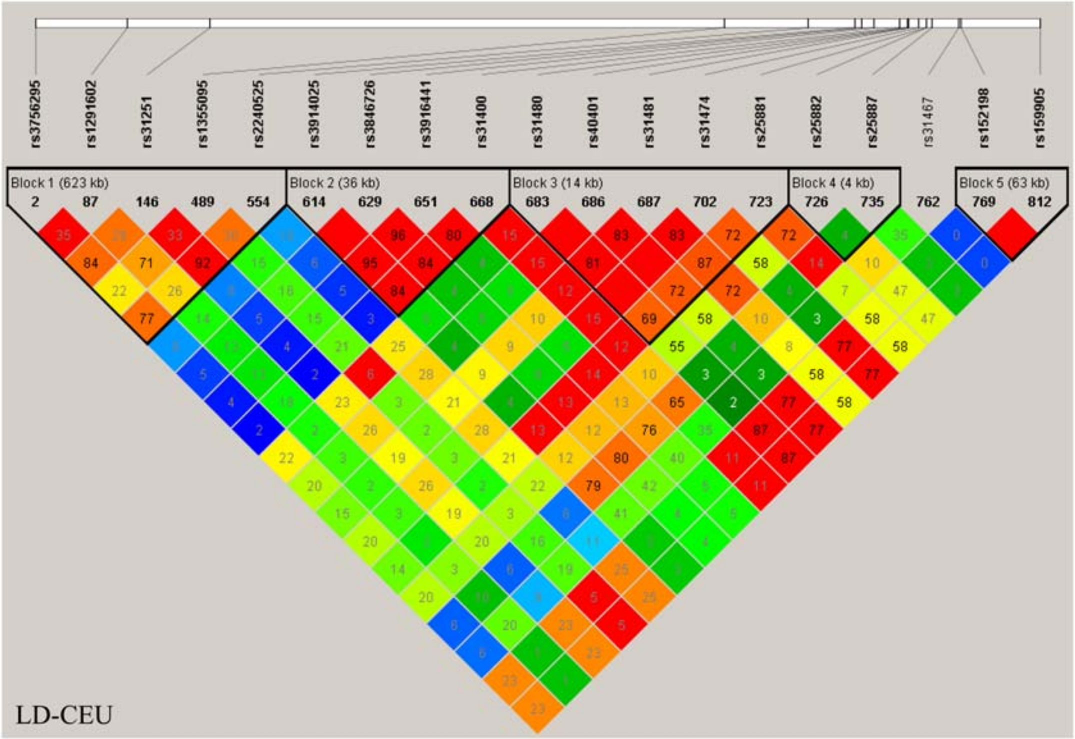

Supplement: Figure S1 — Linkage disequilibrium (LD) pattern of the studies SNPs in Chinese (CHB) and Europeans (CEU). (a) In screening sample (CHB), they are four haplotype blocks and all the 7 highly significant association SNPs are located in block 2. (b) In CEU, they are five haplotype blocks and the 7 highly linked SNPs in CHB are disrupted. LD values (r2) for each pair of markers were calculated by Haploview (v4.2). Haplotype blocks were defined according to the criteria of Gabriel et al. (PDF) [file pone.0050375.s001.pdf]
