# Supplementary figures and images for: The Interleukin 3 Gene (IL3) Contributes to Human Brain Volume Variation by Regulating Proliferation and Survival of Neural Progenitors
Source: PLoS One. 2012 Nov 30;7(11):e50375. doi: 10.1371/journal.pone.0050375 (PMC3511536; doi:10.1371/journal.pone.0050375)

**Figure S2.** The C allele of rs31480 is highly conserved in vertebrate.

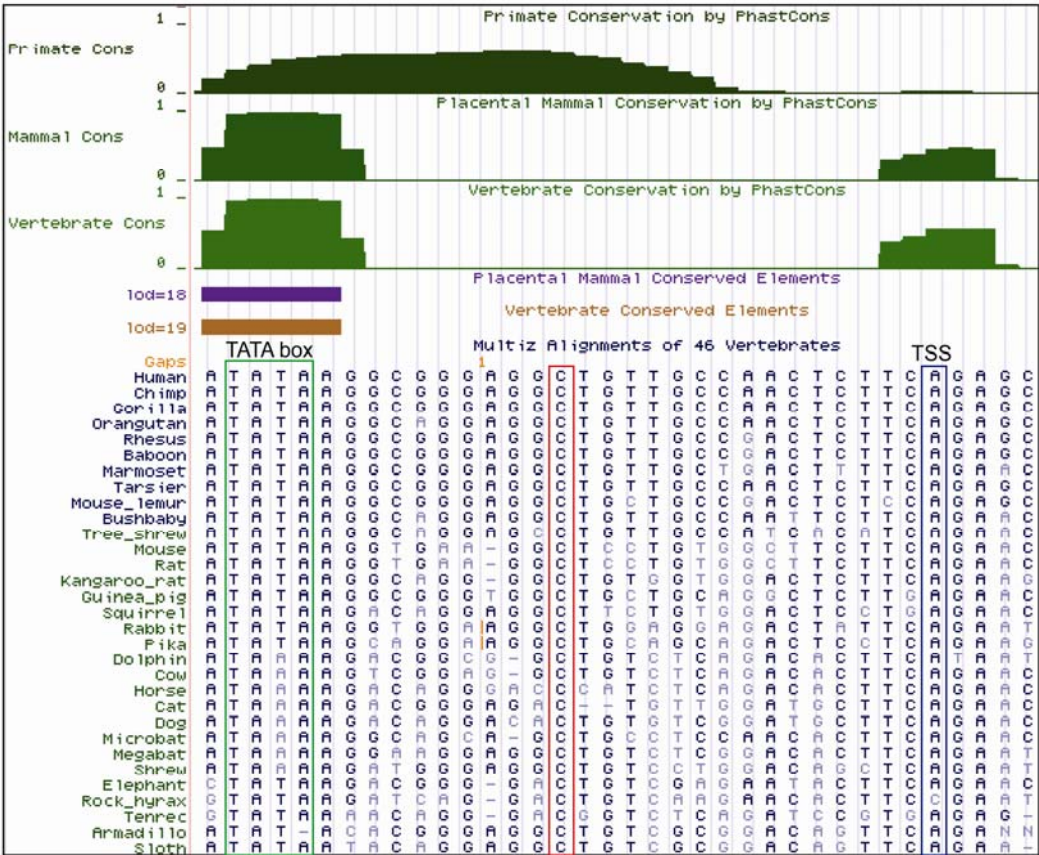

Supplement: Figure S2 — The C allele of rs31480 is highly conserved in vertebrate. rs31480 (box in red) is locates −16 bp upstream of the IL3 promoter, and only 10 bp downstream of the highly conserved TATA binding site (box in blue). The C allele (ancestral allele) is completely conserved in all of the listed species, implying functional importance of rs31480. Note that rs31480 is lies in a primates conserved region (up panel), also suggesting the importance of rs31480 in primates. TSS, transcription start site. (PDF) [file pone.0050375.s002.pdf]

**Figure S3.** Impacts of rs31480 on promoter activity in CHO, SK-N-SH and COS-7 cell lines.

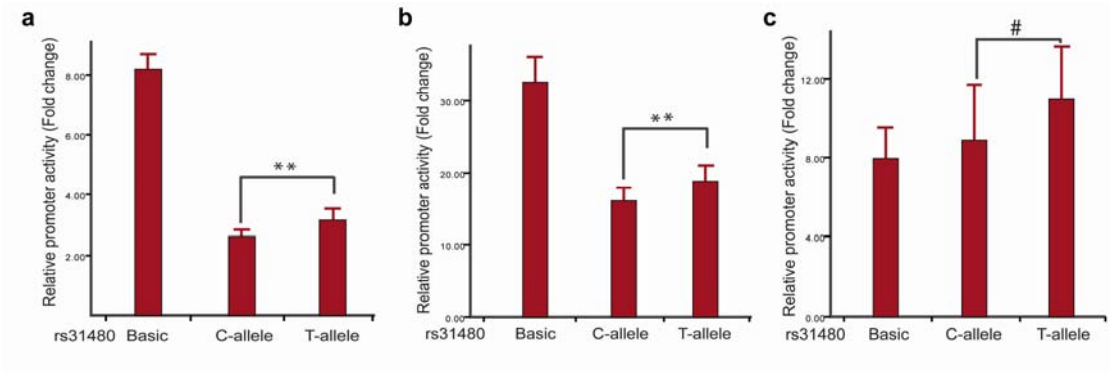

Supplement: Figure S3 — Impacts of rs31480 on promoter activity in CHO, SK-N-SH and COS-7 cell lines. Promoter activity of the construct with T allele is significantly higher than C allele in CHO (a) and COS-7 (b) cells. In SK-N-SH cells (c), the trend is same as CHO and COS-7 though the differences were not reached significant level. Values of relative luciferase activity are expressed as mean ± s.d. (results of a triplicate assay). #P = 0.07, **P<0.005 (Student’s t-test). (PDF) [file pone.0050375.s003.pdf]

**Figure S4.** Expression of IL3RA in embryonic mouse brain (E12.5).

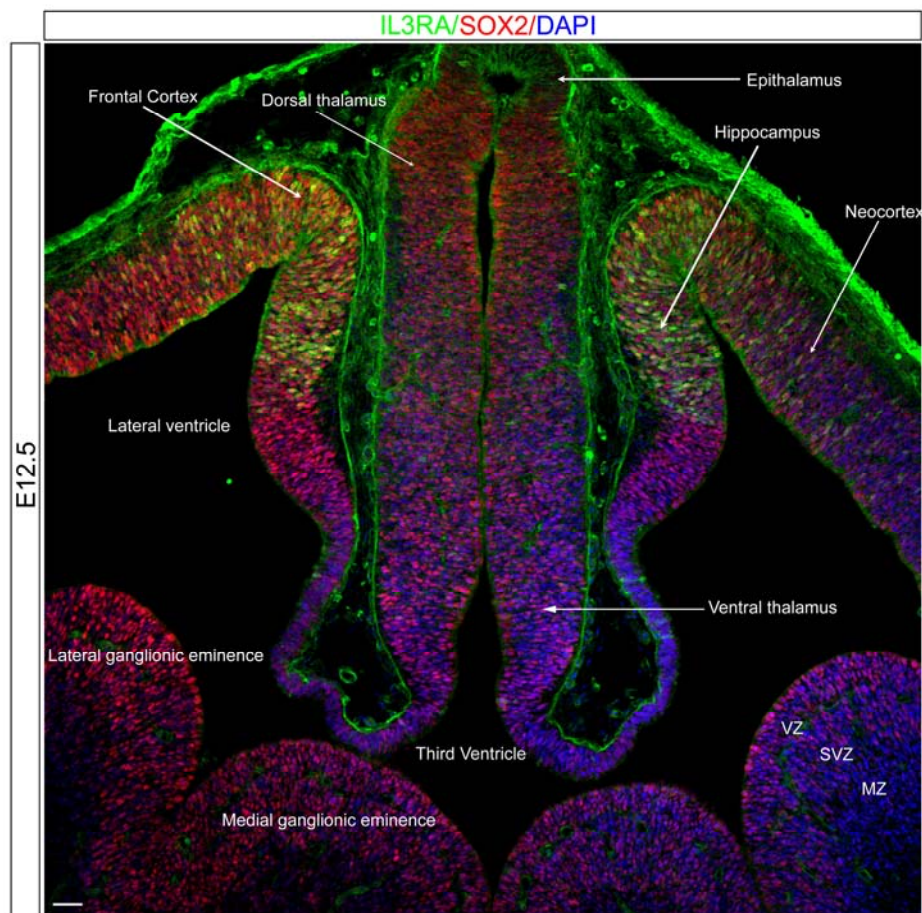

Supplement: Figure S4 — Expression of IL3RA in embryonic mouse brain (E12.5). IL3RA is expressed in SOX2 positive cells in Frontal cortex and hippocampus, two regions that associated with higher level cognitive functions. Scale bar, 50 µm. (PDF) [file pone.0050375.s004.pdf]

**Figure S5.** IL3RA is mainly expressed in the neocortex region of mouse brain.

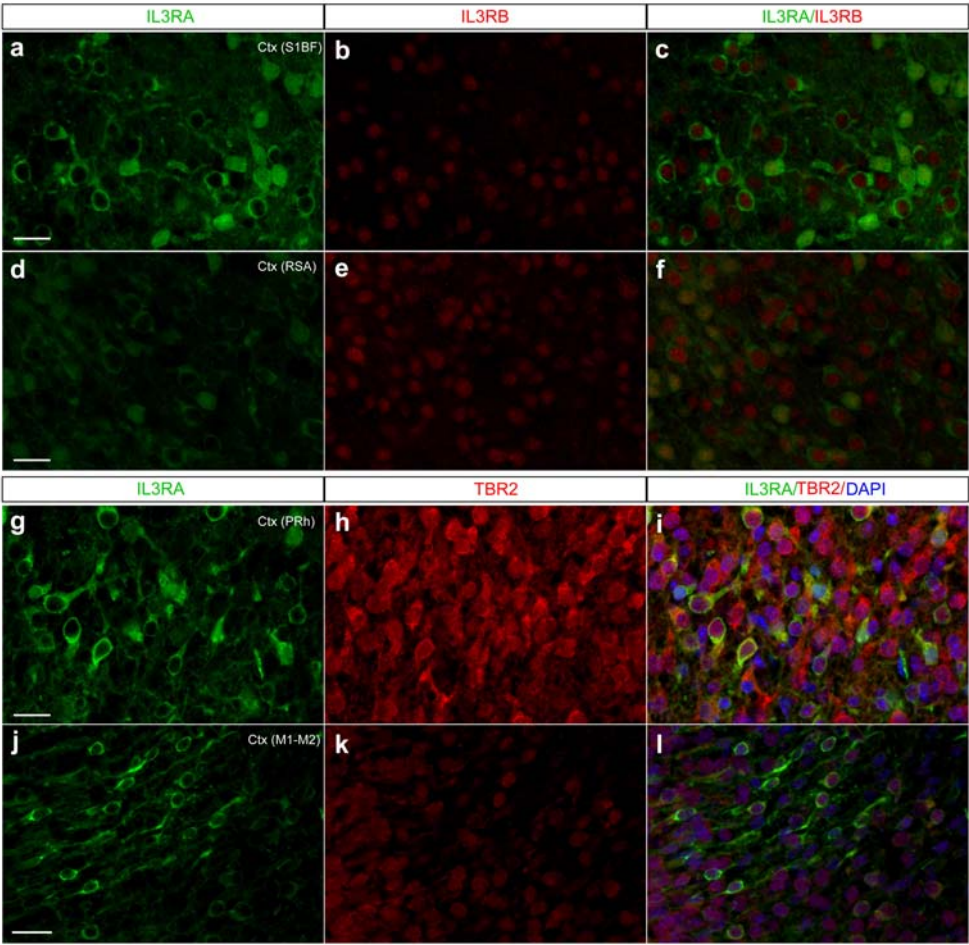

Supplement: Figure S5 — IL3RA is mainly expressed in the neocortex region of mouse brain. (a–f) Co-expression of IL3RA and IL3RB in agranular retrosplenial cortex (RSA), barrel field of the primary somatosensory cortex (S1BF). (g–l) Expression of IL3RA was also found in perirhinal cortex (Prh), primary Motor Cortex (M1) and secondary Motor Cortex (M2). Note that IL3RA positive cells were also expressed Tbr2 weakly, indicating they were not mature neurons. Scale bar, 25 µm. (PDF) [file pone.0050375.s005.pdf]

**Figure S6.** Expression of IL3RA and IL3RB in hippocampus.

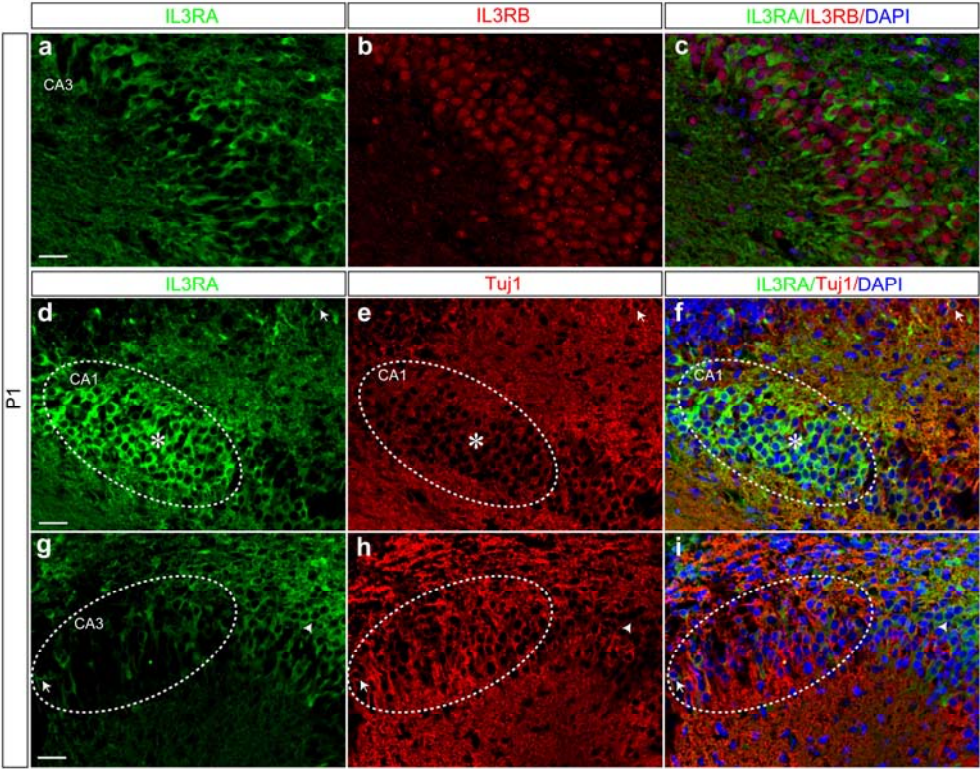

Supplement: Figure S6 — Expression of IL3RA and IL3RB in hippocampus. (a–c) Co-expression of IL3RA and IL3RB in CA3 region of hippocampus. (d–i) IL3RA is expressed in CA1 and CA3 of hippocampus, some of IL3RA positive cells were Tuj1 positive, indicating they were mature neurons, whereas others were Tuj1 negative. Scale bar, 25 µm. (PDF) [file pone.0050375.s006.pdf]

**Figure S7.** IL3RA is expressed in hilus of dentate gyrus.

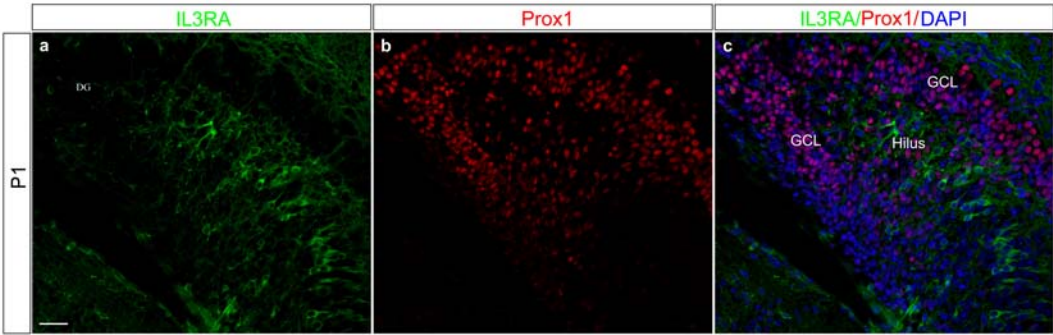

Supplement: Figure S7 — IL3RA is expressed in hilus of dentate gyrus. Prox1 was used to label the granule cell layer (GCL) of the dentate gyrus, note IL3RA positive cells in hilus were prox1 negative. Scale bar, 50 µm. (PDF) [file pone.0050375.s007.pdf]

**Figure S9.** Co-expression of IL3RA and IL3RB in mouse brain.

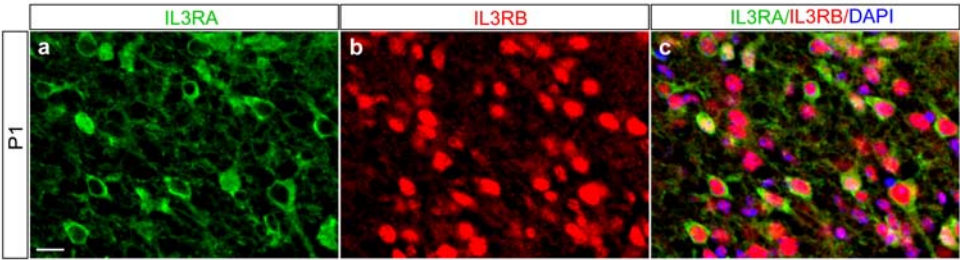

Supplement: Figure S9 — Co-expression of IL3RA and IL3RB in mouse brain. All IL3RA positive cells were also expression IL3RB. Scale bar, 25 µm. (PDF) [file pone.0050375.s009.pdf]

**Figure S10.** IL3RA is mainly expressed in neural progenitors at early embryonic stage.

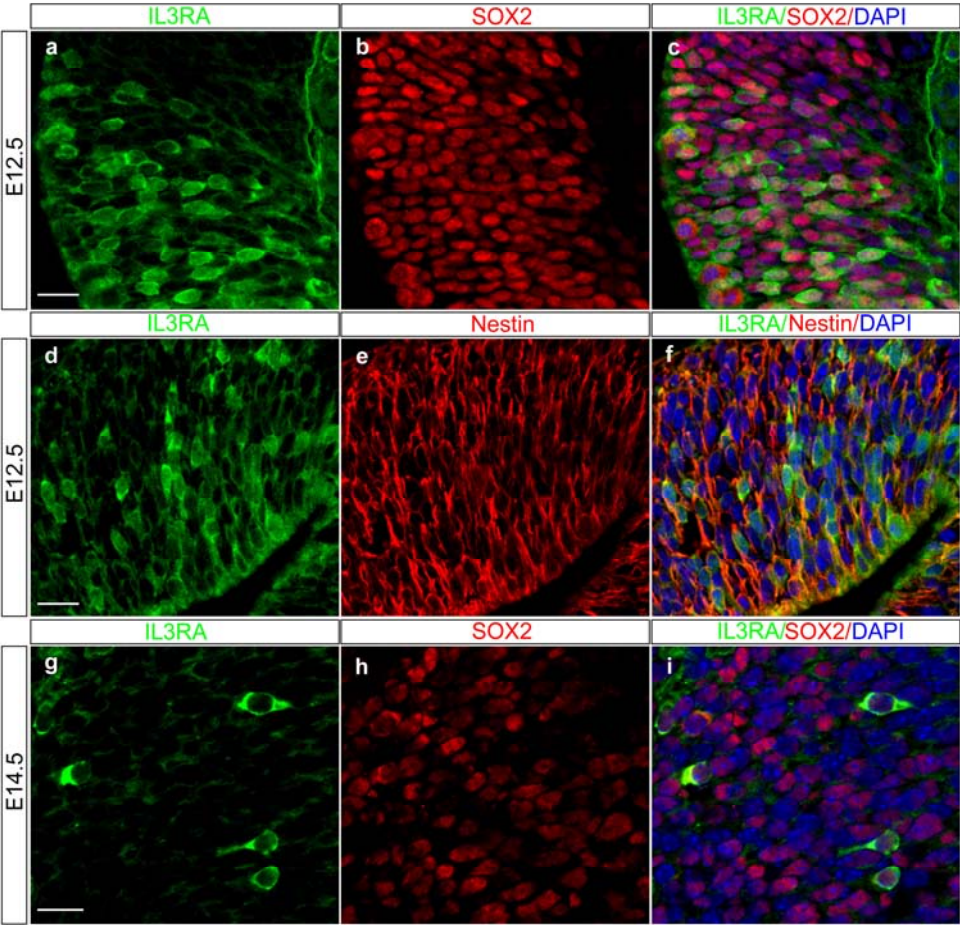

Supplement: Figure S10 — IL3RA is mainly expressed in neural progenitors at early embryonic stage. (a–c) At E12.5, IL3RA is expressed in sox2 positive progenitors in frontal cortex. (b–e) Co-expression of IL3RA and nestin, a marker for neural progenitors. (f–h) At E14.5, IL3RA is expressed in some sox2 positive progenitors in cingulate cortex. Scale bar, 25 µm. (PDF) [file pone.0050375.s010.pdf]

**Figure S11.** Double immunostaining analysis of IL3RA and Tuj1 in the developing mouse brain.

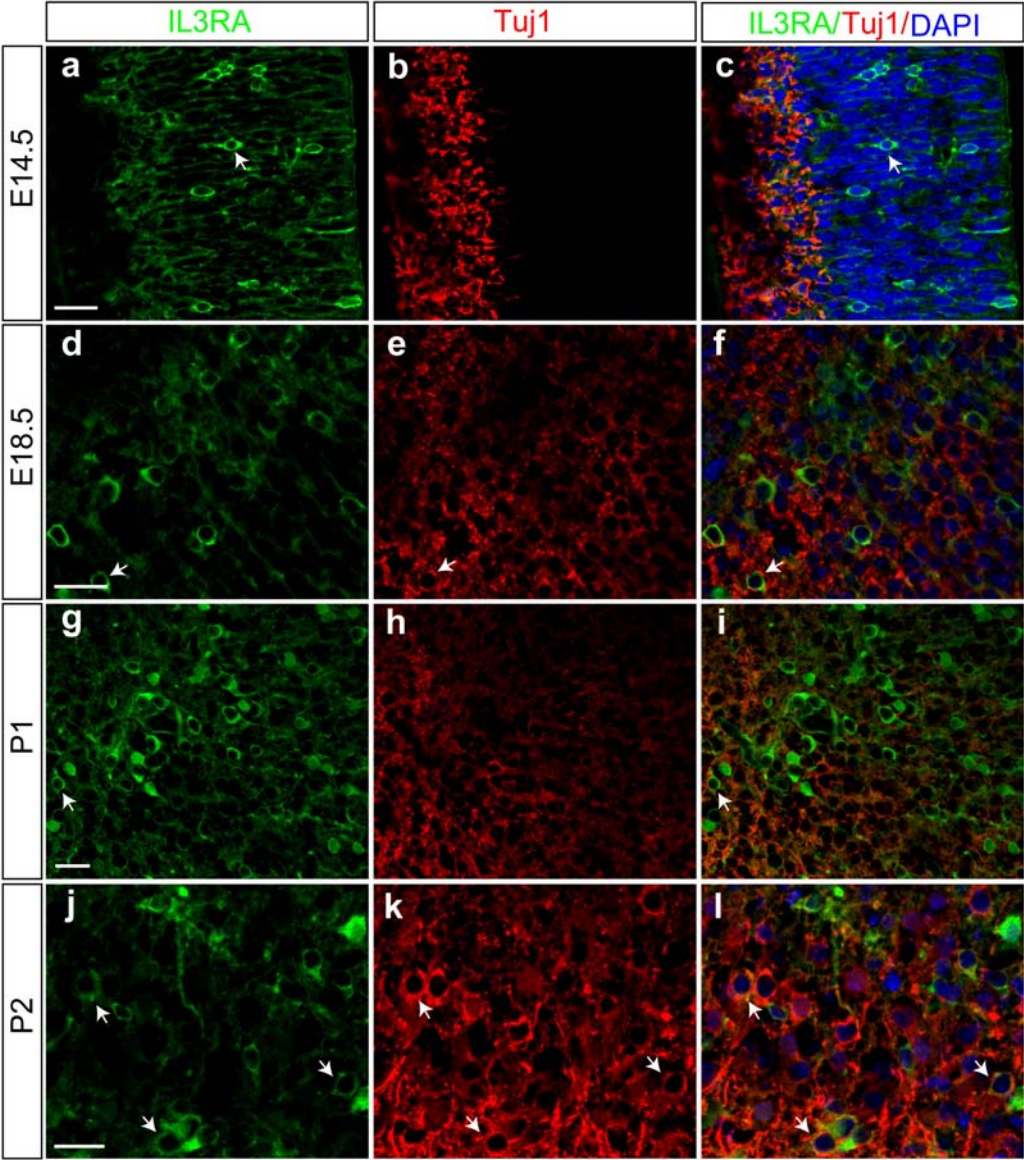

Supplement: Figure S11 — Double immunostaining analysis of IL3RA and Tuj1 in the developing mouse brain. At early stage of brain development (From E14.5-P1), IL3RA is not expressed in mature neurons (a–i). However, at P2 stage, a proportion of IL3RA positive cells are mature neurons as revealed by co-localization with tuj1 (j–l), a marker for mature neurons. Scale bar, 25 µm. (PDF) [file pone.0050375.s011.pdf]

**Figure S12.** IL3RA is expressed in immediate progenitor cells (IPCs).

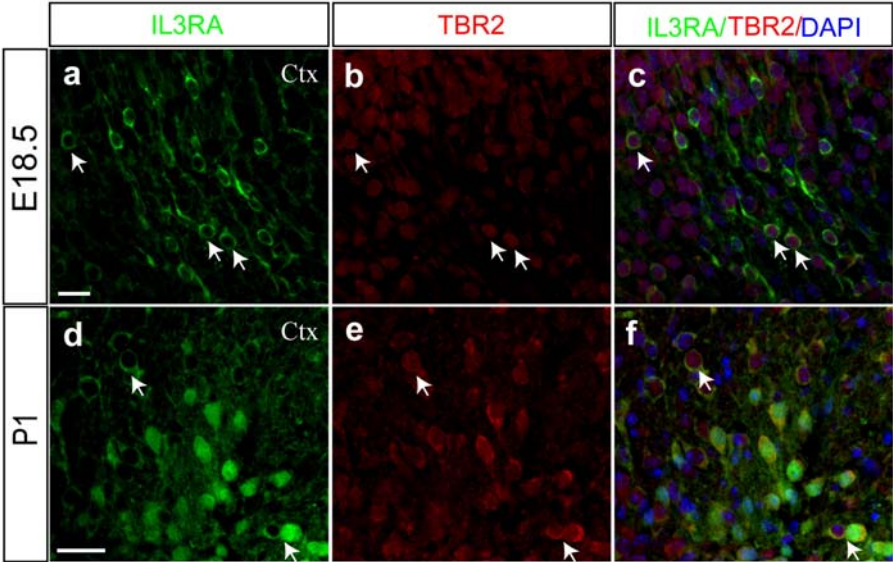

Supplement: Figure S12 — IL3RA is expressed in immediate progenitor cells (IPCs). IL3RA positive cells also express Tbr2 weakly, indicating these cells were immediate progenitors. Scale bar, 25 µm. (PDF) [file pone.0050375.s012.pdf]

**Figure S13.** IL3RB is expressed in neurons and glia cells.

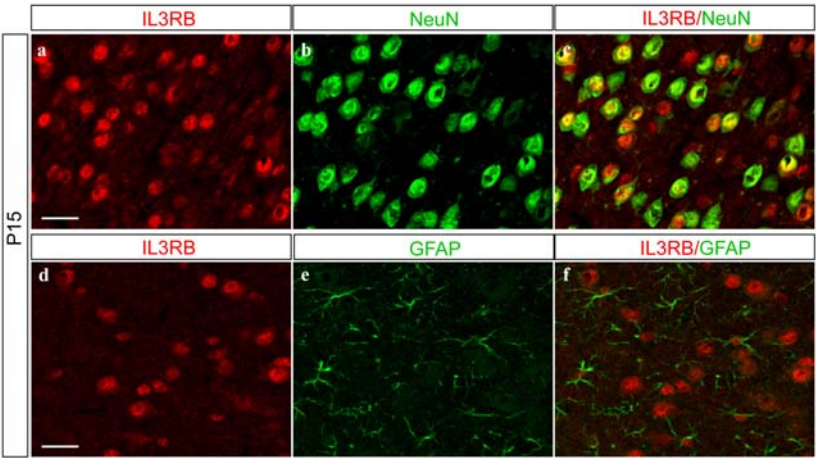

Supplement: Figure S13 — IL3RB is expressed in neurons and glia cells. (a–c) Co-immunofluorescence of IL3RB and NeuN revealed expression of IL3RB in mature neurons. (d–f) IL3RB also expressed in some glia cells (GFAP positive). Scale bar, 25 µm. (PDF) [file pone.0050375.s013.pdf]

**Figure S14.** Expression of IL3RA in proliferating neural progenitors.

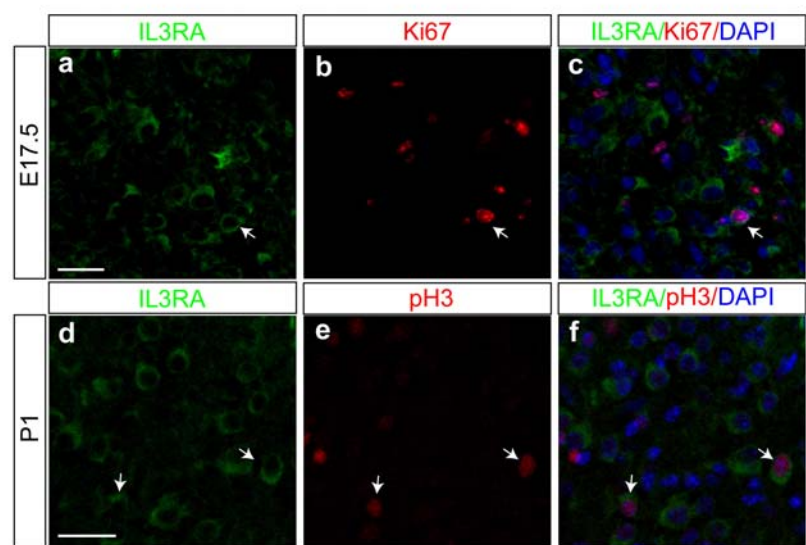

Supplement: Figure S14 — Expression of IL3RA in proliferating neural progenitors. Some IL3RA positive cells also expressed Ki67 and pH3, marker for proliferation cells, indicating they were active proliferation. Scale bar, 25 µm. (PDF) [file pone.0050375.s014.pdf]

**Figure S16.** IL3 has no effects on neural differentiation.

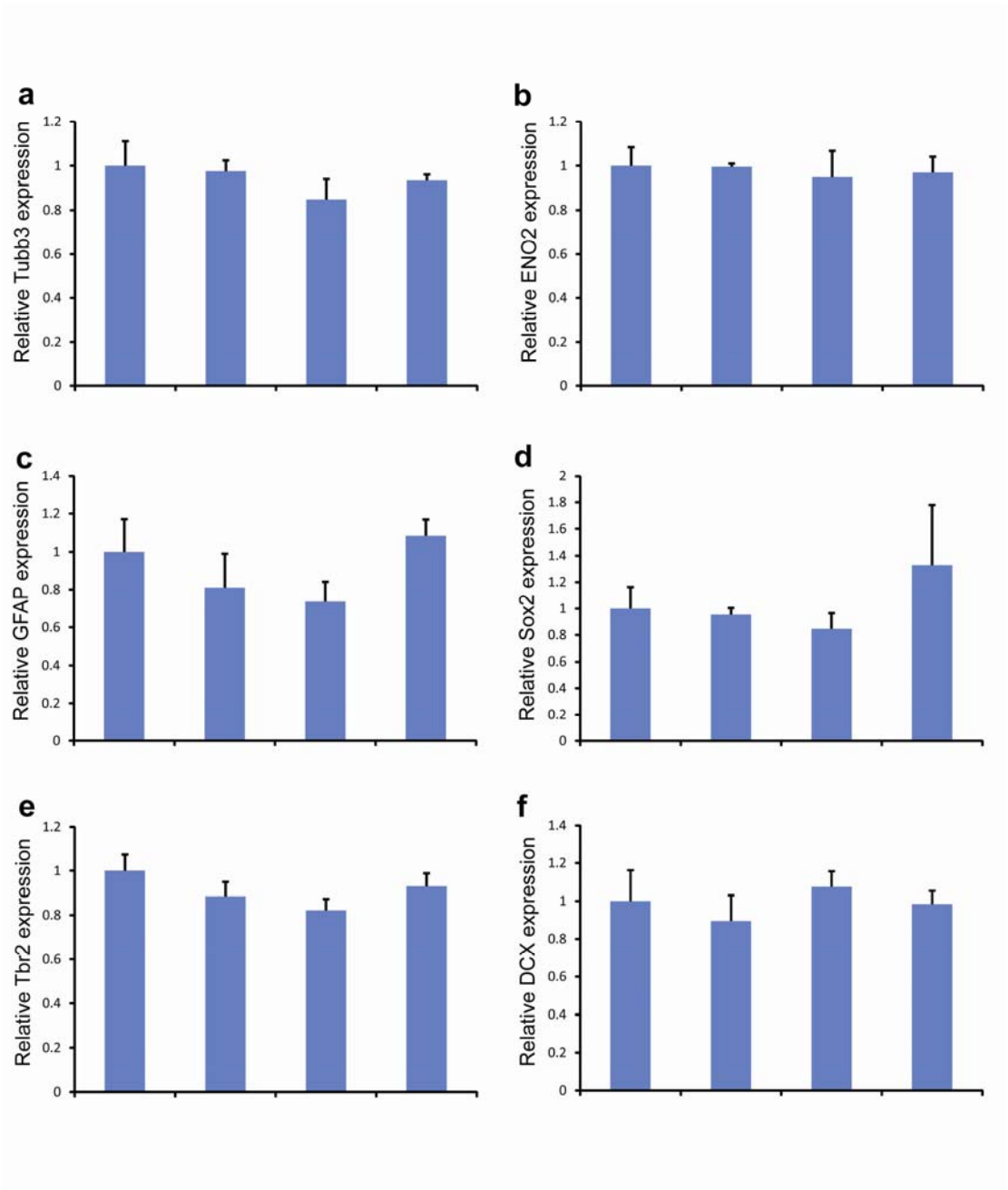

Supplement: Figure S16 — IL3 has no effects on neural differentiation. Neural progenitors were first cultured in neurobasal medium under proliferation condition (containing 10 ng/ml FGF2 and EGF), after 4 day’s culture, FGF2 and EGF were removed. Then 2% FBS and different concentrations of IL3 (10, 100, 200 ng/ml) were added. The cultures were maintained for 4 days and RNA was isolated for quantification. Relative gene expression was not changed for all of the tested genes, indicating IL3 has no effect on neural differentiation. Real-time PCR analysis of beta-III Tubulin (Tubb3) (a) and Enolase 2 (ENO2) (b), two neuron specific markers, GFAP (glia cells marker) (c), sox2 (neural stem cells marker) (d), Tbr2 (immediate progenitors marker) (e), and DCX (new born neurons marker) (f). None of these cell specific markers showed significant change after different concentration IL-3 treatment. (PDF) [file pone.0050375.s016.pdf]

**Figure S17.** IL-3 is not regulated by estrogen.

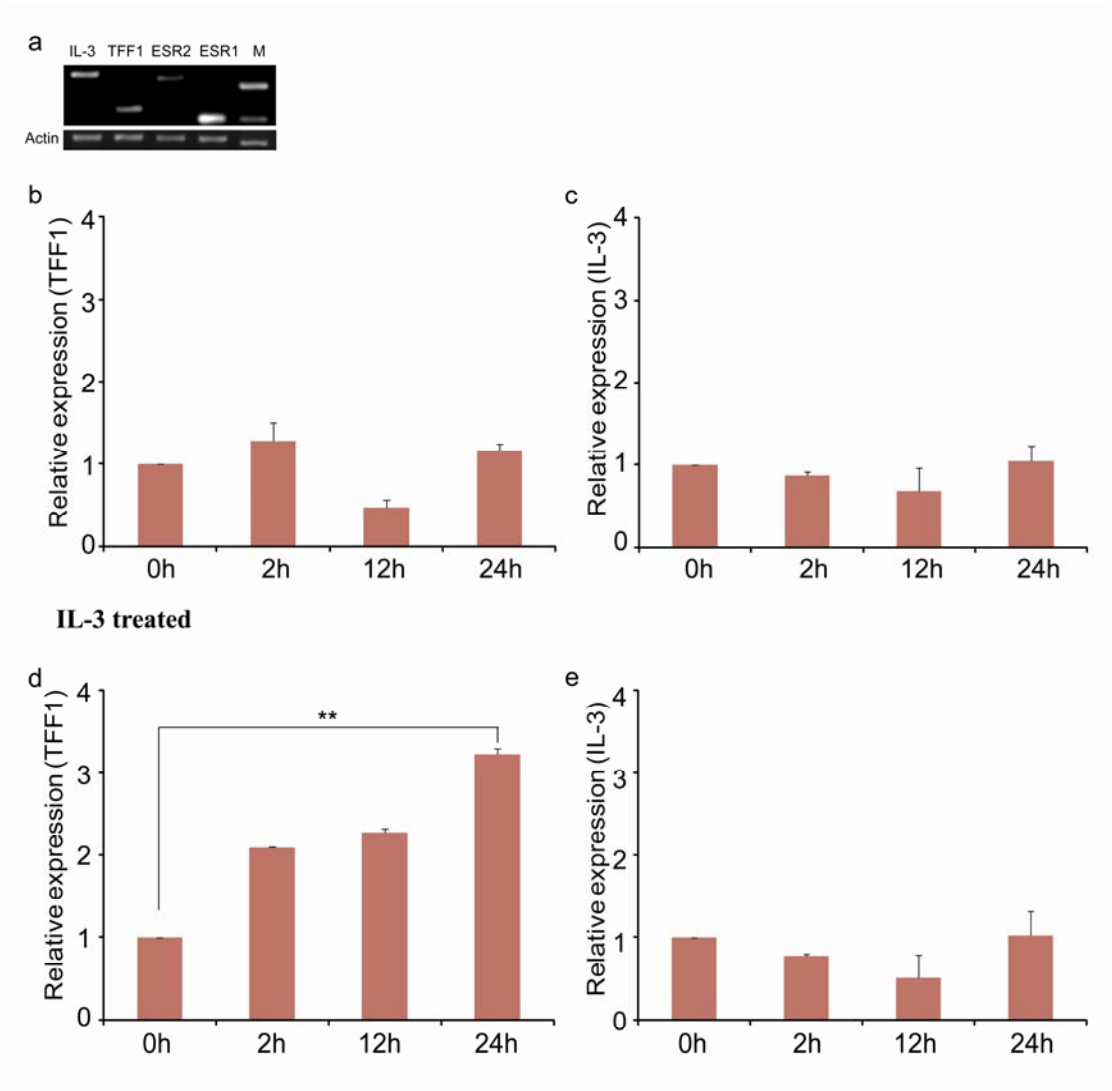

Supplement: Figure S17 — IL-3 is not regulated by estrogen. We first confirmed that K562 cell line expressed IL3 and estrogen receptors (ESR1 and ESR2) by RT-PCR (a). (b–c) Expression of TFF1 and IL3 was not changed after treated by vehicle (DMSO) for different times (0 h–24 h). TFF1, an estrogen response gene, showed significantly elevated after treated by estrogen (10 nM) (d), however, expression of IL-3 was not changed after estrogen treatment (e), indicating IL-3 is not regulated by estrogen. Data are expressed as mean ± s.e.m. (three independent assays, each containing 3 replicates). **P<0.01. (PDF) [file pone.0050375.s017.pdf]

**Figure S18.** Model for sex-specific association of IL-3 and brain volume.

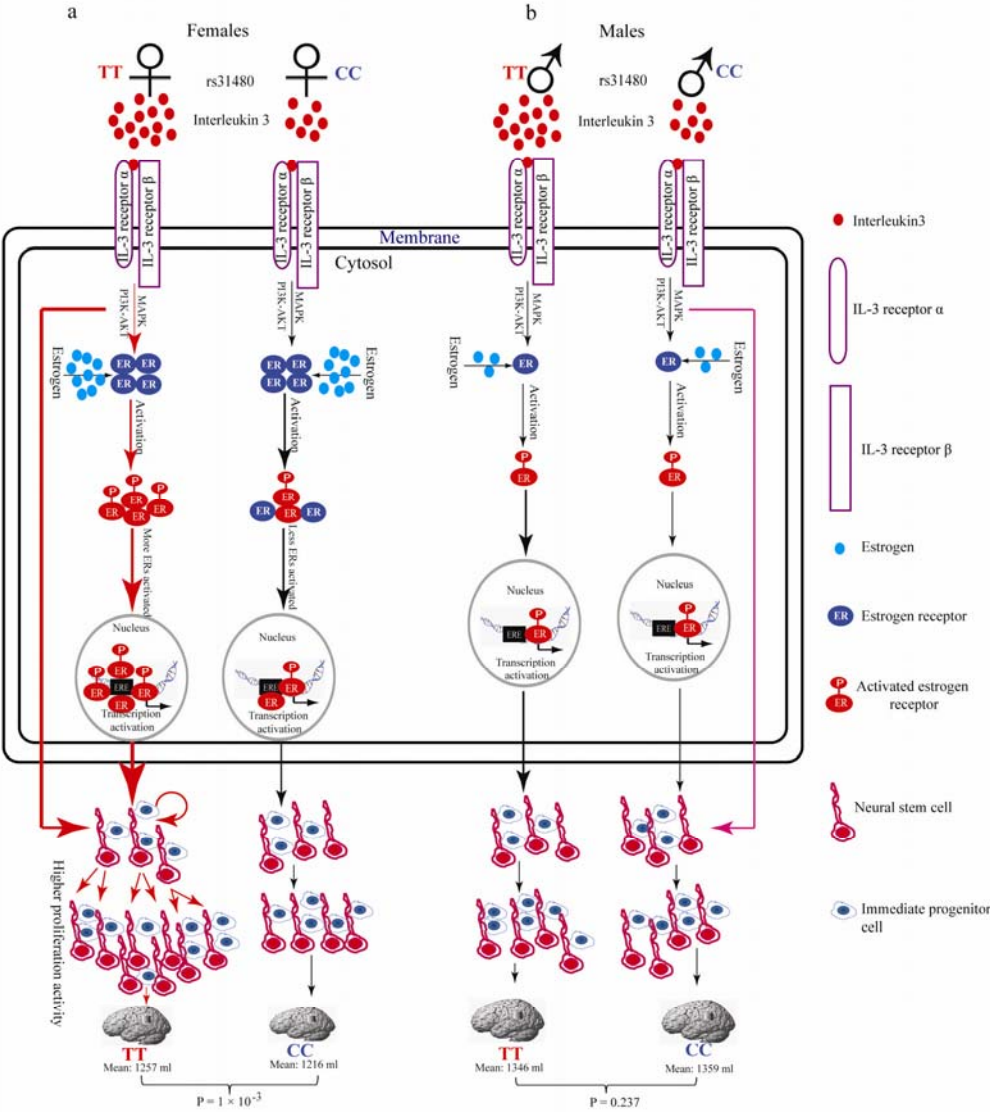

Supplement: Figure S18 — Model for sex-specific association of IL-3 and brain volume. Different genotypes at rs31480 (TT vs. CC) influence IL-3′s expression in both males and females. However, since the estrogen receptors (ER) level is low in males, therefore, even the signaling pathways mediated by IL-3 were different in TT and CC carriers, the total activation level of ERs was not significant changed. But in females, the activation level of ERs was different between TT and CC carriers due to high level of ERs. In addition, estrogen can further enhance ER activity in females. As a result, signaling pathways mediated by ER were greatly activated in TT carriers than in CC carriers, which may influence brain development, eventually lead to difference of brain volume in TT and CC carriers at rs31480. (PDF) [file pone.0050375.s018.pdf]
