# Supplementary material for: The Interleukin 3 Gene (IL3) Contributes to Human Brain Volume Variation by Regulating Proliferation and Survival of Neural Progenitors
Source: PLoS One. 2012 Nov 30;7(11):e50375. doi: 10.1371/journal.pone.0050375 (PMC3511536; doi:10.1371/journal.pone.0050375)

Figure S8. Double immunostaining of IL3RA and SOX2 revealed co-localization of IL3RA and sox 2 in developing mouse brain.

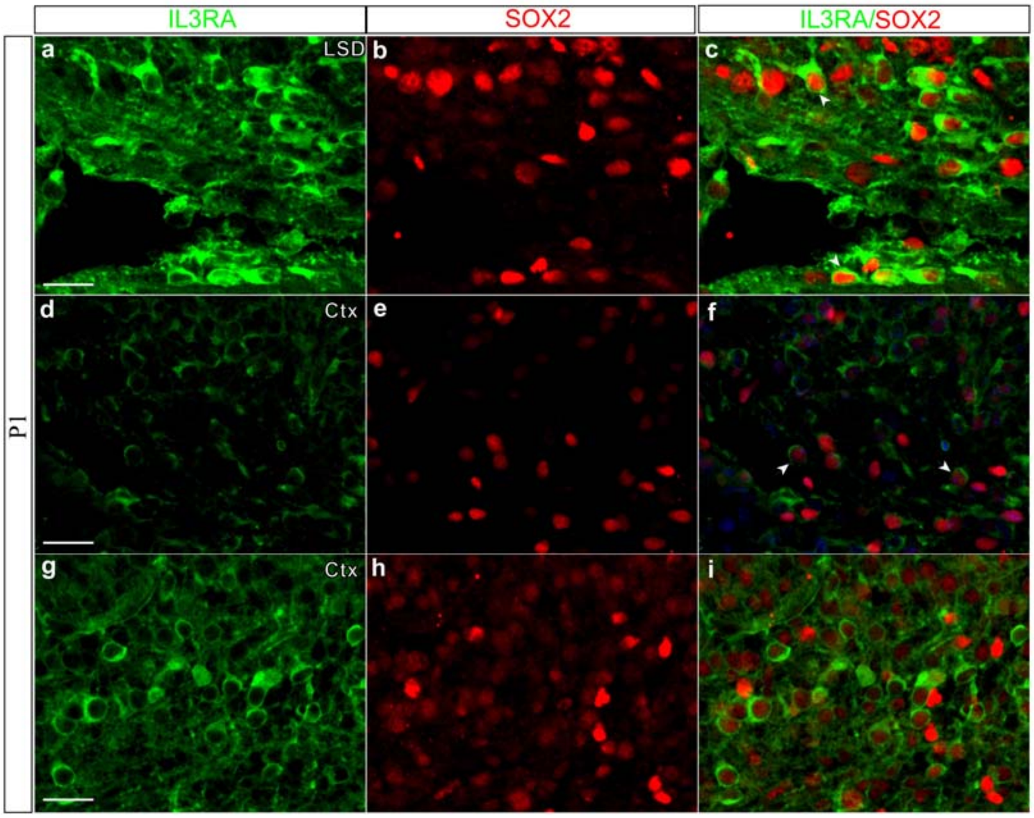

Supplement: Figure S8 — Double immunostaining of IL3RA and SOX2 revealed co-localization of IL3RA and SOX2 in developing mouse brain. (a–c) IL3RA expression cells were SOX2 positive in lateral septal nucleus, dorsal part (LSD), indicating they were neural progenitors. (d–i) In cortex, some IL3RA positive cells still express SOX2, but for many of IL3RA positive cells, expression of SOX2 was down-regulated or turned-off, demonstrating they were converted into immediate progenitors or neurons. Scale bar, 25 µm. (PDF) [file pone.0050375.s008.pdf]
