# Supplementary material for: The Interleukin 3 Gene (IL3) Contributes to Human Brain Volume Variation by Regulating Proliferation and Survival of Neural Progenitors
Source: PLoS One. 2012 Nov 30;7(11):e50375. doi: 10.1371/journal.pone.0050375 (PMC3511536; doi:10.1371/journal.pone.0050375)

Figure S15. Expression of IL3 receptors (IL3RA and IL3RB) in cultured neural progenitors and trophic effects of IL-3 on neural progenitors and neurons.

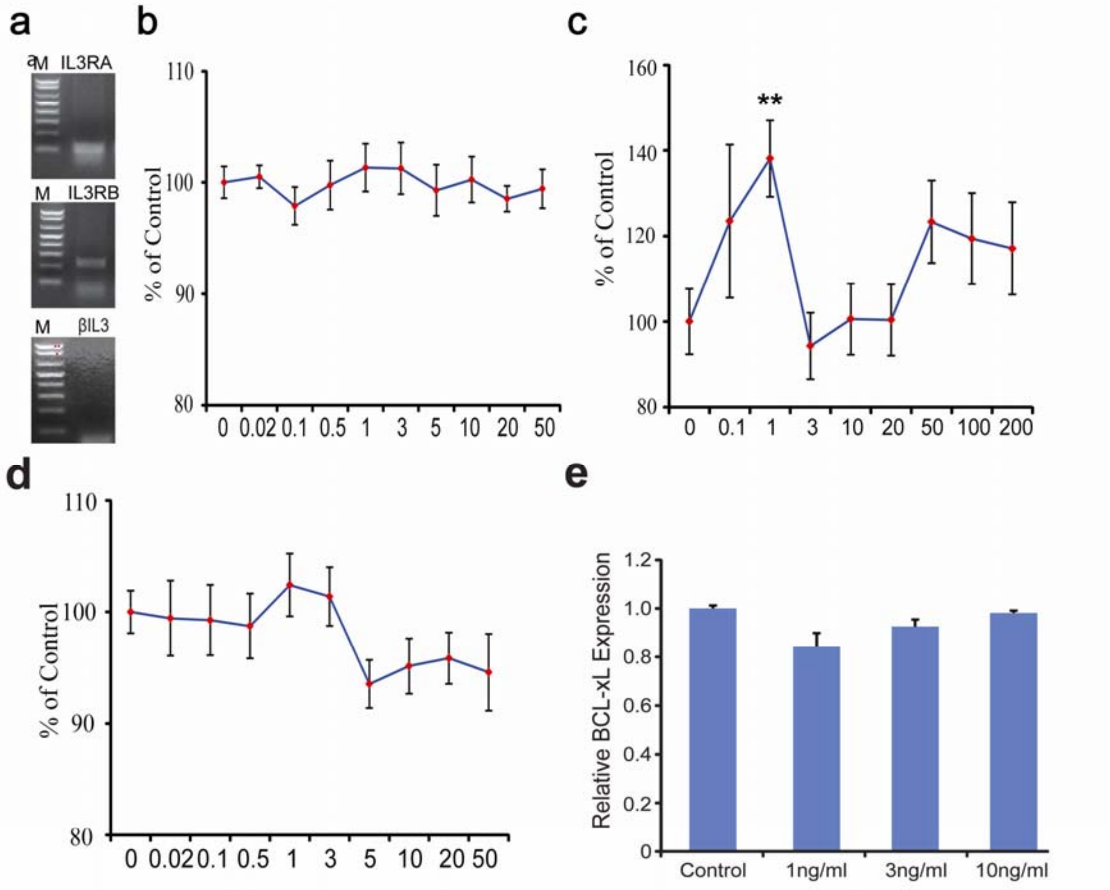

Supplement: Figure S15 — Expression of IL3 receptors (IL3RA and IL3RB) in cultured neural progenitors and trophic effects of IL-3 on neural progenitors and neurons. (a) IL3 receptors (IL3RA and IL3RB) were expressed in cultured neural progenitors (from E13.5 mice) as revealed by RT-PCR, however, βIL3 was not detected. (b) When there were 5% FBS in neurobasal medium, IL-3 has no trophic effects on neural progenitors. Neural progenitors from E12.5 mice were cultured in neurobasal medium (supplemented with 5% FBS and IL-3) for 36 hours then cell viability was measured. (c) IL-3 (1 ng/ml) significantly promotes survival of neurons when there were no any factors in neurobasal medium. Neurons from E17.5 mice were cultured in neurobasal medium (supplemented with B27 and Glutamax) for 12 days, then B27 and Glutamax were removed from medium and different concentrations of IL-3 were added. Cell viability was determined after IL-3 treatment for 2 days. (d) However, when there were B27 supplement in neurobasal medium, trophic effects of IL-3 on neurons was disappeared. Neurons from E18 mice were first cultured in neurobasal medium (supplemented with B27 and different concentrations of IL-3) for 24 hours, cell viability was then measured. Y-axis, cell viability (normalized to control); X-axis, concentration of IL-3 (ng/ml). Data are expressed as mean ± s.e.m. (n = 8 for each group). *P<0.05 (Student’s t-test). (e) The expression of BCL-xL was not regulated by IL3 in neural progenitors. (PDF) [file pone.0050375.s015.pdf]
