# Supplementary material for: The Interleukin 3 Gene (IL3) Contributes to Human Brain Volume Variation by Regulating Proliferation and Survival of Neural Progenitors
Source: PLoS One. 2012 Nov 30;7(11):e50375. doi: 10.1371/journal.pone.0050375 (PMC3511536; doi:10.1371/journal.pone.0050375)
Supplement: Table S1 — Marker characteristics and association significance in females. (DOC) [file pone.0050375.s019.doc]

**Table S1. Marker characteristics and association significance in females**

| Marker | Location | polymorphism | R^2^ | T^a^ | P value^a^ (Original) | P value^b^ (Corrected) |
| --- | --- | --- | --- | --- | --- | --- |
| **rs3756295** | 130720739 | G/C | 0.008146 | -1.994 | **0.04674** | 0.9348 |
| **rs40396** | 130735943 | G/C | 0.01127 | 2.329 | **0.02028** | 0.4056 |
| **rs1291602** | 130794561 | G/A | 1.725e-005 | -0.09157 | 0.9271 | 1.000 |
| **rs31251** | 130861845 | G/A | 0.01574 | -2.787 | **0.00552** | 0.1104 |
| **rs1355095** | 131276668 | G/A | 7.536e-005 | -0.1908 | 0.8488 | 1.000 |
| **rs2240525** | 131343783 | C/T | 0.01304 | -2.523 | **0.01194** | 0.2388 |
| **rs3914025** | 131381184 | G/A | 0.02725 | 3.64 | **0.0003028** | **0.006056** |
| rs3846726 | 131386898 | G/A | 0.02978 | -3.858 | **0.0001295** | **0.00259** |
| rs3916441 | 131397140 | C/T | 0.03173 | -4.044 | **6.086e-005** | **0.001217** |
| **rs31400** | 131417406 | G/A | 0.03161 | -3.962 | **8.542e-005** | **0.001708** |
| rs31480 | 131424231 | G/A | 0.02412 | 3.519 | **0.0004728** | **0.009456** |
| rs40401 | 131424377 | G/A | 0.02707 | 3.734 | **0.0002104** | **0.004208** |
| rs31481 | 131425101 | C/T | 0.02715 | 3.702 | **0.0002381** | **0.004762** |
| rs31474 | 131432926 | G/A | 0.02145 | -3.317 | **0.0009748** | **0.01950** |
| rs25881 | 131439037 | G/A | 0.007004 | 1.861 | 0.06334 | 1.000 |
| rs25882 | 131439395 | G/A | 0.005714 | -1.676 | 0.09432 | 1.000 |
| rs25887 | 131443960 | G/T | 0.006902 | 1.858 | 0.06369 | 1.000 |
| rs31467 | 131464737 | G/A | 0.00501 | 1.588 | 0.1129 | 1.000 |
| rs152198 | 131466709 | G/A | 0.001003 | 0.7072 | 0.4798 | 1.000 |
| rs159905 | 131530402 | G/A | 0.001421 | 0.8393 | 0.4017 | 1.000 |

^a^The test statistics T and P values were obtained by Plink v1.06 under the additive genetic model. The bold font markers indicated these markers were genotyped in the first stage first in screening sample. For fine scale mapping, the other markers were genotyped. ^b^The corrected P values were generated by using Bonferroni correction. Statistically significant p-values are displayed in bold.
