# Supplementary material for: The Interleukin 3 Gene (IL3) Contributes to Human Brain Volume Variation by Regulating Proliferation and Survival of Neural Progenitors
Source: PLoS One. 2012 Nov 30;7(11):e50375. doi: 10.1371/journal.pone.0050375 (PMC3511536; doi:10.1371/journal.pone.0050375)
Supplement: Table S2 — Core haplotype association analysis in females. (DOC) [file pone.0050375.s020.doc]

**Table S**2. Core haplotype association analysis in females

| rs3914025 | rs3846726 | rs3916441 | rs31400 | rs31480 | rs40401 | rs31481 | Hap-Freq | Hap-Score^a^ | p-val^b^ | simp-val^c^ |
| --- | --- | --- | --- | --- | --- | --- | --- | --- | --- | --- |
| G | A | T | A | G | G | C | 0.46054 | -3.35407 | **8.00E-04** | 4.00E-04 |
| A | G | C | G | G | G | C | 0.00802 | -0.90253 | 0.36677 | 0.36105 |
| A | G | C | G | A | A | C | 0.00604 | -0.79477 | 0.42674 | 0.42872 |
| G | A | T | G | G | G | C | 0.00729 | -0.75395 | 0.45088 | 0.44728 |
| A | G | T | A | G | G | C | 0.00589 | -0.73046 | 0.46511 | 0.46903 |
| A | G | C | A | A | A | T | 0.00726 | -0.55135 | 0.58139 | 0.58115 |
| A | G | T | G | A | A | T | 0.00641 | -0.51225 | 0.60847 | 0.59277 |
| G | A | T | A | G | G | T | 0.00561 | -0.04123 | 0.96711 | 0.9653 |
| G | G | C | A | A | A | T | 0.0066 | -0.00485 | 0.99613 | 0.99546 |
| A | G | C | G | A | A | T | 0.46458 | 4.08904 | **4.00E-05** | 0 |

^a^The Hap-scores were obtained by Haplo.stats v1.3.1.^34^ The positive values indicate positive correlation with cranial volume, while the negative values indicate negative correlation with cranial volume; ^b^P-val, the P-value calculated from the Hap-score; ^c^sim P-value, the P-value calculated by simulation; core haplotypes : the haplotypes consisting of the 7 highly significant association SNPs. Statistically significant p-values are displayed in bold.
