# Supplementary material for: The Interleukin 3 Gene (IL3) Contributes to Human Brain Volume Variation by Regulating Proliferation and Survival of Neural Progenitors
Source: PLoS One. 2012 Nov 30;7(11):e50375. doi: 10.1371/journal.pone.0050375 (PMC3511536; doi:10.1371/journal.pone.0050375)
Supplement: Table S3 — Marker characteristics and association significance in males. (DOC) [file pone.0050375.s021.doc]

**Table S3. Marker characteristics and association significance in males**

| Marker | Location | polymorphism | R^2^ | T | P value |
| --- | --- | --- | --- | --- | --- |
| rs3756295 | 130720739 | G/C | 0.0002783 | 0.3337 | 0.7388 |
| rs40396 | 130735943 | G/C | 0.00114 | -0.6647 | 0.5066 |
| rs1291602 | 130794561 | G/A | 3.762e-005 | 0.1228 | 0.9023 |
| rs31251 | 130861845 | G/A | 0.00163 | 0.8121 | 0.4172 |
| rs1355095 | 131276668 | G/A | 0.0004007 | -0.3979 | 0.6909 |
| rs2240525 | 131343783 | C/T | 0.002468 | 0.9949 | 0.3204 |
| rs3914025 | 131381184 | G/A | 0.001773 | -0.8387 | 0.4022 |
| rs3846726 | 131386898 | G/A | 0.005633 | 1.502 | 0.134 |
| rs3916441 | 131397140 | C/T | 0.002404 | 0.9964 | 0.3197 |
| rs31400 | 131417406 | G/A | 0.001008 | 0.6329 | 0.5272 |
| rs31480 | 131424231 | G/A | 0.003637 | -1.22 | 0.2231 |
| rs40401 | 131424377 | G/A | 0.002816 | -1.073 | 0.2837 |
| rs31481 | 131425101 | C/T | 0.00094 | -0.6104 | 0.5419 |
| rs31474 | 131432926 | G/A | 0.0004788 | 0.4421 | 0.6586 |
| rs25881 | 131439037 | G/A | 0.003949 | -1.25 | 0.2121 |
| rs25882 | 131439395 | G/A | 0.004317 | 1.307 | 0.192 |
| rs25887 | 131443960 | G/T | 0.00284 | -1.078 | 0.2817 |
| rs31467 | 131464737 | G/A | 0.01146 | -2.17 | 0.03062 |
| rs152198 | 131466709 | G/A | 0.0007232 | -0.5447 | 0.5862 |
| rs159905 | 131530402 | G/A | 1.021e-006 | -0.02041 | 0.9837 |
