# Supplementary material for: The Interleukin 3 Gene (IL3) Contributes to Human Brain Volume Variation by Regulating Proliferation and Survival of Neural Progenitors
Source: PLoS One. 2012 Nov 30;7(11):e50375. doi: 10.1371/journal.pone.0050375 (PMC3511536; doi:10.1371/journal.pone.0050375)
Supplement: Table S4 — Average cranial volumes of female individuals with three different genotypes at each of the seven SNPs covering IL-3. (DOC) [file pone.0050375.s022.doc]

**Table S4. Average cranial volumes of female individuals with three different genotypes at each of the seven SNPs covering IL-3**

| **Marker** | **Average Brain Volume ± SD (ml)** | | |
| --- | --- | --- | --- |
| rs3914025 | AA(1252 ± 94) | AG(1226 ± 91) | GG(1208 ± 92) |
| rs3846726 | AA(1209 ± 94) | AG(1226 ± 90) | GG(1254 ± 94) |
| rs3916441 | TT(1210 ± 94) | TC(1226 ± 91) | CC(1257 ± 91) |
| rs31400 | AA(1209 ± 90) | AG(1225 ± 93) | GG(1256 ± 92) |
| rs31480 | TT(1257 ± 92) | TC(1224 ± 89) | CC(1216 ± 98) |
| rs40401 | AA(1257 ± 92) | AG(1224 ± 89) | GG(1213 ± 98) |
| rs31481 | TT(1254 ± 93) | TC(1225 ± 89) | CC(1211 ± 98) |
