# Supplementary material for: The Interleukin 3 Gene (IL3) Contributes to Human Brain Volume Variation by Regulating Proliferation and Survival of Neural Progenitors
Source: PLoS One. 2012 Nov 30;7(11):e50375. doi: 10.1371/journal.pone.0050375 (PMC3511536; doi:10.1371/journal.pone.0050375)
Supplement: Table S5 — Marker characteristics of AKT1 and association significance. (DOC) [file pone.0050375.s023.doc]

**Table S5. Marker characteristics of AKT1 and association significance**

| Marker | Location | polymorphism | Minor allele | MAF | P value | |
| --- | --- | --- | --- | --- | --- | --- |
|  |  |  |  |  | females | males |
| rs2494732 | AKT1 | C/T | T | 0.296 | 0.895 | 0.113 |
| rs10149779 | AKT1 | G/A | A | 0.081 | 0.011 | 0.566 |
| rs3803300 | AKT1 | C/T | C | 0.310 | 0.039 | 0.957 |
